# Protective effects of the R-(+)-thioctic acid treatment: possible anti-inflammatory activity on heart of hypertensive rats

**DOI:** 10.1186/s12906-024-04547-6

**Published:** 2024-07-24

**Authors:** Proshanta Roy, Daniele Tomassoni, Ilenia Martinelli, Vincenzo Bellitto, Giulio Nittari, Francesco Amenta, Seyed Khosrow Tayebati

**Affiliations:** 1 School of Pharmacy, University of Camerino, Via Madonna Delle Carceri, 9, Camerino, 62032 MC Italy; 2 School of Biosciences and Veterinary Medicine, University of Camerino, Via Gentile III da Varano, Camerino, 62032 MC Italy

**Keywords:** Hypertension, Oxidative stress, Inflammation, Heart, Thioctic acid

## Abstract

**Background:**

In cardiovascular disease, high blood pressure is associated with oxidative stress, promoting endothelial dysfunction, vascular remodeling, and inflammation. Clinical trials are discordant that the most effective treatment in the management of hypertension seems to be the administration of anti-hypertensive drugs with antioxidant properties. The study aims to evaluate the effects of the eutomer of thioctic acid on oxidative stress and inflammation in the heart of spontaneously hypertensive rats compared to normotensive Wistar Kyoto rats.

**Methods:**

To study the oxidative status, the malondialdehyde and 4-hydroxynonenal concentration, protein oxidation were measured in the heart. Morphological analysis were performed. Immunohistochemistry and Western blot were done for alpha-smooth muscle actin and transforming growth factor beta to assess fibrosis; cytokines and nuclear factor kappaB to assess inflammatory processes.

**Results:**

Spontaneously hypertensive rats were characterized by hypertension with increased malondialdehyde levels in the heart. OxyBlot in the heart of spontaneously hypertensive rats showed an increase in proteins’ oxidative status. Cardiomyocyte hypertrophy and fibrosis in the ventricles were associated with an increased expression of alpha-smooth muscle actin and pro-inflammatory cytokines, reduced by the eutomer of thioctic acid supplementation.

**Conclusions:**

Based on this evidence, eutomer of thioctic acid could represent an appropriate antioxidant molecule to reduce oxidative stress and prevent inflammatory processes on the cardiomyocytes and cardiac vascular endothelium.

**Supplementary Information:**

The online version contains supplementary material available at 10.1186/s12906-024-04547-6.

## Introduction

Hypertension or high blood pressure (HBP), a chronic medical condition in which the arterial blood pressure is persistently elevated [1], represents an important public health problem [2]. HBP constitutes the major risk factor for coronary and peripheral arterial diseases, heart failure, stroke, atrial fibrillation, vision loss, chronic kidney disease, and cerebrovascular alterations associated with dementia [3]. The involvement of oxidative stress in cardiovascular damage related to vascular remodeling, endothelial dysfunction, and inflammation was previously reported [4]. The increased bioavailability of reactive oxygen species (ROS) related to mitochondrial oxidative stress contributes to hypertensive cardiomyocyte damage development [5, 6]. The pathogenesis and progression associated with cardiovascular diseases consist in the inflammation of the endothelial lining tunica intima, the initiation of oxidative stress in the arterial wall, the thickening of the blood vessel and subsequently plaque formation in the arteries [7–9].

Complex interacting mechanisms that direct vascular smooth muscle activity includes the renin-angiotensin-aldosterone system, sympathetic nervous innervation, immunological action, and oxidative stress [10]. The upregulation of the renin-angiotensin-aldosterone system, the sympathetic nervous system activation, the impairment of the G protein-coupled receptor signaling, the altered T-cell activity and inflammation have all been linked to the pathogenesis of hypertension [11]. Alterations of cardiomyocytes and other resident cells of the myocardium (fibroblasts, pericytes, endothelial and immune cells) and recruitment of immune and inflammatory cells and progenitor cells from the circulation lead to a complex process known as myocardial remodeling [12–14]. Increased bioavailability of ROS is a common feature of this process, as are increased nitric oxide (NO) levels and impaired antioxidant capacity in the cardiovascular, renal, and neurological systems [15, 16]. Oxidative stress is fundamental in the establishment of hypertension. The biomarkers of oxidative stress are increased in patients with hypertension, and oxidative damage is correlated with the molecular mechanisms of cardiovascular injury in hypertension [17, 18]. Several clinical trials disagree that the most effective treatment in managing hypertension is the administration of anti-hypertensive drugs with antioxidant properties [19].

Oxidative stress activates different transcription factors, which lead to the differential expression of genes involved in inflammatory pathways [20]. The inflammation triggered by oxidative stress with the activation of the pro-inflammatory molecules like tumor necrosis factor-alpha (TNF-alpha), interleukin-1 beta (IL-1 beta) and interleukin-6 (IL-6), and cell adhesion molecules such as E-selectin has an important role in the pathogenesis of vascular remodeling. Consequently, remodeling causes vascular stiffness and rises the blood pressure [21–23].

Thioctic acid (TIO) is a naturally occurring antioxidant compound that comes in two optical isomers [24]. The naturally occurring R-enantiomer (+)-TIO is the dextrorotatory form, whereas the most commonly utilized formulation in clinical practice is a combination of (+) and (-) enantiomers [25, 26]. (+)-TIO acts as a cofactor for several important mitochondrial multienzyme complexes; enhances the uptake of glucose; modulates the transcription factors and activates various signaling pathways. It was shown that TIO and its reduced form have a direct antioxidant effect due to the neutralization of ROS that is destructive to DNA, proteins, and lipids of cells [27]. (+)-TIO is synthesized in the human body and is contained in foods in a form covalently associated with lysine (lipoyllysine) [27].

Even if TIO demonstrated a powerful antioxidant activity in vitro, a short half-life and a modest oral bioavailability were found in vivo [28]. Indeed, therapeutic efficacy is relatively low due to its pharmacokinetic limitation (hepatic degradation, reduced solubility, and gastric instability). However, liquid preparations of TIO and new amphiphilic matrices formulations have significantly enhanced TIO bioavailability and, consequently, its therapeutic efficacy [25]. Better pharmacokinetic parameters were found in the (+)-TIO [26]. The advantage in using (+)-TIO conjugated with lysine salt compared to the racemic form may be linked to an amplified bioavailability and biological activity of this enantiomer that enhanced the antioxidant activity as shown both in vitro [29] and in vivo studies [30–33].

We previously demonstrated (+)-TIO as an appropriate antioxidant molecule to reduce oxidative stress, cardiac alterations [30], and adhesion molecule expression in the vascular endothelium of spontaneously hypertensive rats (SHR) [31]. SHR is a rat strain used in assessing hypertensive-related end-organ damage and its possible treatment. This is possibly related to the effects at the levels of the endothelial walls of vessels that determine vasodilation [34].

Left ventricular cardiomyocyte hypertrophy, the deposition of reticulin and collagen fibers, protein oxidation, IL-1 beta, IL-6, and TNF-alpha expression were analyzed on the heart of SHR treated or not with (+)-TIO. The SHR were compared to the age matched normotensive Wistar Kyoto rats (WKY). The results aim to suggest this molecule as a possible antioxidant to prevent heart injury associated with hypertension.

## Methods

### Animals handling, treatment and tissue processing

SHR develop spontaneously arterial hypertension [35], and are used as an animal model of heart failure because its progression of cardiac remodeling toward heart failure is reportedly like that seen in humans [36]. Animals used for these experiments belonged to a wider study focused on the protective effects of antioxidants on cerebrovascular alteration in SHR. This study was designed according to the ARRIVE (Animal Research: Reporting of In Vivo Experiments) guideline and approved by the Ministry of Health based on the D.lgs 26/2014 (Authorization n°163/2019-PR February 25, 2019) after the acceptance of the Committee “Organismo Preposto al Benessere degli Animali” of University of Camerino. Rats were handled according to internationally accepted principles for the care of laboratory animals (European Community Council Directive 86/609, O.J. n° L358, Dec. 18, 1986). The sample size defined for the experiments on the brain allowed us to maintain the test power up to 90% for the parameters analyzed in the heart (Statistical software, Origin 9.1).

Male SHR aged 20 weeks (*n* = 16) and age-matched WKY rats (*n* = 8) were used and randomized to have homogenous features regarding to weight and blood pressure. SHR were treated (*n* = 8) or not (*n* = 8) for 4 weeks with 125 μmol/kg/day of (+)-TIO lysine salt (Sintactica, Servizi Chimico Farmaceutici, Lotto RALA. L 1,911,261) solubilized in physiologic solution and intraperitoneally administered [32]. The (+)-TIO is the enantiomer R that represents the active form of the racemic compound [30–32]. Control WKY and SHR rats received the same amounts of vehicle. Rats were housed 1 per cage under constant temperature (22–24 °C), and a 12 h light/dark cycle (light on at 07:00), food, and water were available ad libitum. Food and water intake were monitored daily.

Once a week, systolic and diastolic blood pressures were measured by a tail cuff equipped with a photoelectric pulse detector in conscious SHR and WKY rats. The body weights were weekly taken. Animals were anesthetized with isoflurane and then perfused. The heart was dissected out, weighed and divided in two parts. One was fixed for 72 h in 4% paraformaldehyde, and processed for the paraffin wax embedding after alcohol dehydration and clarification in xylene. The second part was immediately frozen at -80 °C for biochemical analysis.

### Assay of thiobarbituric acid reactive substances

Lipid peroxidation was quantified in heart homogenates by measuring the accumulation of thiobarbituric acid reactive substances (TBARS) (Cayman, Chemical Company, Ann Arbor, MI, USA, Cat. No. 10009055) and expressed as malondialdehyde (MDA) content [31, 37]. The amount of MDA was measured spectrophotometrically at 532 nm, following the datasheet of the company.

### Western blot

The tissue was homogenized with lysis buffer to obtain proteins as previously described [30, 37]. After the protein concentration measurement by Bradford assay (Bio-Rad, Hercules, CA, USA), 40 μg of proteins were separated through Sodium Dodecyl Sulphate-Polyacrylamide Gel Electrophoresis (SDS-PAGE) and transferred to nitrocellulose membranes [30, 37]. The membranes were blocked for 1 h using 5% bovine serum albumin (BSA) and non-fat dry milk in phosphate-buffered saline (PBS) containing Tween 20 and were then probed overnight at 4 °C using one of the primary antibodies as detailed in Table 1. Then, the membranes were transferred at room temperature and blotted for 1 h at room temperature with horseradish peroxidase (HRP)-conjugated secondary antibodies (Bethyl Laboratories, Inc., Montgomery, TX, USA, dilution 1:5000). The detection of band intensities was performed using the Lite blot Plus or Turbo kits (Euroclone, Milan, Italy). The optical density was determined through Bio-Rad image analysis (Bio-Rad, Hercules, CA, USA). For quantification, β-actin was used as a protein loading control. Also, the protein oxidation status was investigated in the heart using the OxyBlot Protein Oxidation Detection kit (Merck Millipore, Burlington, MA, USA, Cat. No. S7150), according to the manufacturer’s instructions.

**Table 1:** Antibodies’ dilution for Immunohistochemistry (IHC) and Western Blot (WB) analysis

Antibodies	Company	Dilution IHC	Dilution WB
4-Hydroxynonenal (4-HNE)	Santa Cruz Biotechnology sc-130083	/	1:500
Alpha-smooth muscle actin (alpha-SMA)	Sigma-Aldrich A2547	1:100	1:300
Transforming growth factor-beta 1 (TGF-beta 1)	Merck-Millipore SAB4502954	/	1:500
E-Selectin	Santa Cruz Biotechnology sc-14011	1:50	1:500
Intercellular adhesion molecule-1 (ICAM-1)	Santa Cruz Biotechnology sc-7891	/	1:500
Vascular cell adhesion molecule-1 (VCAM-1)	Santa Cruz Biotechnology sc-8304	/	1:500
Platelet endothelial cell adhesion molecule-1 (PECAM-1)	Santa Cruz Biotechnology sc-1506	/	1:500
Tumor necrosis factor-alpha (TNF-alpha)	Bio-Rad AAR33	1:750	1:5000
Interleukin-1 beta (IL-1 beta)	Bio-Rad AAR15G	1:750	1:5000
Interleukin-6 (IL-6)	GeneTex GTX110527	1:200	1:2000
Nuclear factor kappa-light-chain-enhancer of activated B cells (NF-kB) p50	Santa Cruz Biotechnology sc-114	1:50	1:100
Beta-actin (β-actin)	Merck-Millipore A2228	/	1:3000

### Morphological analysis and immunohistochemistry

Longitudinal consecutive heart sections were cut using a rotary microtome (Leica RM 2145) and processed for morphological and immunohistochemistry (IHC) analysis as described previously [30, 31, 38]. Sections of the heart were stained with Masson’s trichrome to evaluate cardiomyocytes hypertrophy and fibrosis [30, 38]. Silver impregnation (Diapath S.p.A., Martinengo, BG, Italy, Cat. N. 010211) and Sirius red (Direct Red 80, Sigma Aldrich Cat. N. 365548) staining were performed to highlight the reticulin and collagen fibers deposition, respectively. In addition, sections were processed for IHC analysis as previously described [30, 31, 38] using different antibodies at various dilutions in PBS + TritonX-100 0.3% (PBS-T), as detailed in Table 1. Optimal working concentrations for the antibodies were established through preliminary experiments. After incubation overnight with primary antibodies, slides were exposed for 30 min at 25 °C to the specific biotinylated secondary antibodies (Bethyl Laboratories, Inc., Montgomery, TX, USA) diluted 1:200 in PBS-T. The immunoreaction was revealed after the incubation with an avidin-biotin complex (Vector Laboratories, Inc., Burlingame, CA, USA) and consequently using 3,3′-diamonobenzidine tetrahydrochloride (DAB) solution as a substrate (Vector Laboratories, Inc., Burlingame, CA, USA). The sections were observed using a microscope Leica DMR light microscope connected by a DS-Ri2 NIKON camera to Nikon Image analyzer Software (NIS-Elements, Nikon, Florence, Italy) to record the mean intensity of immune reaction as previously described [30, 38]. For confocal microscopy, the sections were incubated with primary antibodies followed by secondary conjugated Alexa Fluor 594, for 1 h at 37 °C, and then counterstained with 4′,6-diamidino-2-phenylindole (DAPI). Slides were observed with a Nikon mod. C2 plus Confocal Laser Microscope (Nikon, Corporation, Japan). Representative pictures were captured at 40× magnification. Mean fluorescence intensity was measured with the Nikon NIS Element software [38].

### Statistical analysis

Data from the WKY, SHR, and SHR (+)-TIO groups were compared using one-way ANOVA to examine groups differences. Tukey’s multiple-sample comparison test was used to identify appropriate differences. Data are expressed as means ± S.D. A *p*-value less than 0.05 was taken as a minimum level of significance between groups.

## Results

### Blood pressure

**Fig. 1:**
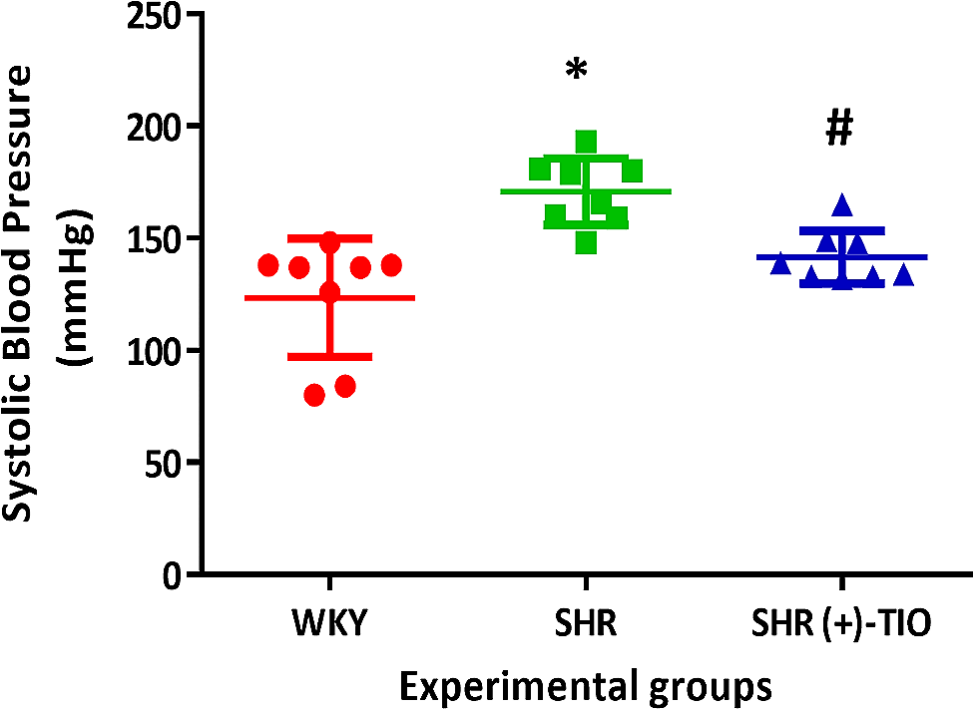
Blood pressure modulation. Systolic blood pressure values in normotensive Wistar Kyoto rats (WKY), spontaneously hypertensive rats (SHR), and SHR treated with (+)-thioctic acid lysine salt [SHR (+)-TIO]. Data, expressed in mmHg, are the mean ± S.D. (*n* = 8/group) *= *p* < 0.05 vs. WKY: #= *p* < 0.05 vs. SHR

Starting 23rd week of age, the systolic blood pressure was significantly higher in SHR groups compared to the normotensive WKY (data not shown). Therefore, the treatment with (+)-TIO started at 24th week of age and lasted for 4 weeks. The systolic blood pressure, measured on the day of sacrifice decreased in the SHR treated-group, compared to the control (Fig. 1). The body weight of hypertensive rats did not change compared to the age-matched WKY, while the heart weight was significantly higher in SHR compared to WKY (Supplementary Fig. 1). Moreover, the heart weight did not change in the (+)-TIO treated group (Supplementary Fig. 1).

### Oxidative stress

**Fig. 2:**
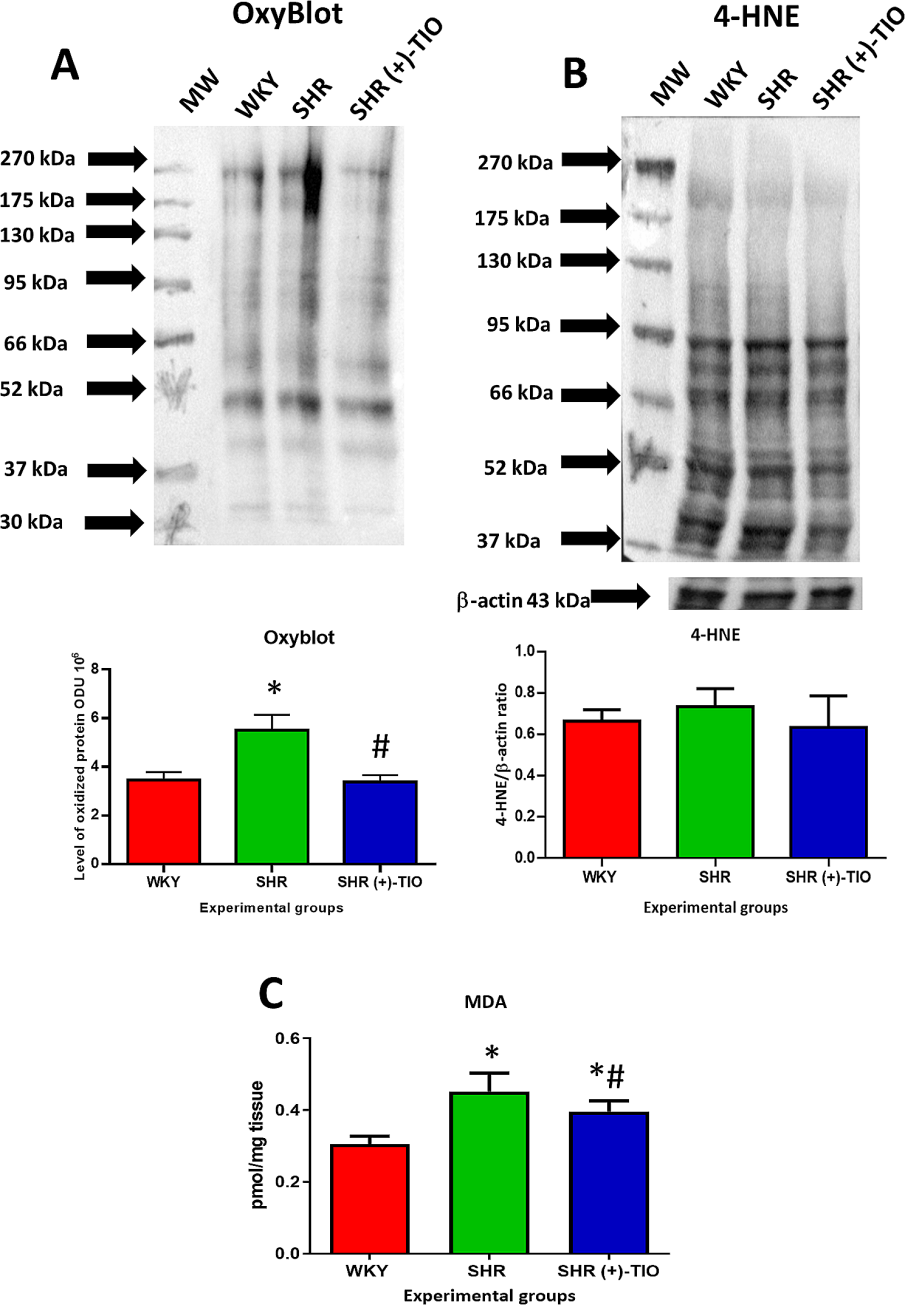
Parameters of oxidative stress in the heart parenchyma. Samples of the heart of normotensive Wistar Kyoto rats (WKY), spontaneously hypertensive rats (SHR), and SHR treated with (+)-thioctic acid lysine salt [SHR (+)-TIO] were immunoblotted with OxyBlot (**A**) and with specific anti-4-hydroxynonenal (4-HNE) antibody (**B**). For the OxyBlot analysis, the bar graph reports the values of optical density measured in the optical density unit (ODU). 4-HNE bar graph indicates the ratio of densitometric analysis of bands to β-actin levels used as the reference loading control. (**C**) Concentration of malondialdehyde (MDA) expressed in pmol/mg of tissue. Data are the mean ± S.D. = *p* < 0.05 vs. WKY: #= *p* < 0.05 vs. SHR

The results of the OxyBlot kit showed an increase of oxidized proteins in the heart parenchyma (Fig. 2) of SHR compared to normotensive WKY rats suggesting that protein oxidation was related to hypertensive status. Treatment with antioxidant (+)-TIO slightly decreased the level of oxidized proteins, in the heart parenchyma (Fig. 2). Associated with the increase in the oxidative status of proteins the heart showed a slight increase in the levels of the 4-hydroxynonenal (4-HNE) (Fig. 2). The supplementation of (+)-TIO did not modify the level of 4-HNE in the heart parenchyma (Fig. 2). The TBARS kit revealed in the heart of SHR an increase in the level of MDA, indicating an increase of lipid peroxidation that was decreased by (+)-TIO supplementation (Fig. 2). The data of increased pro-oxidative elements revealed in SHR an oxidative stress condition related to hypertension, that was counteracted by the (+)-TIO supplementation.

### Morphological aspects

**Fig. 3:**
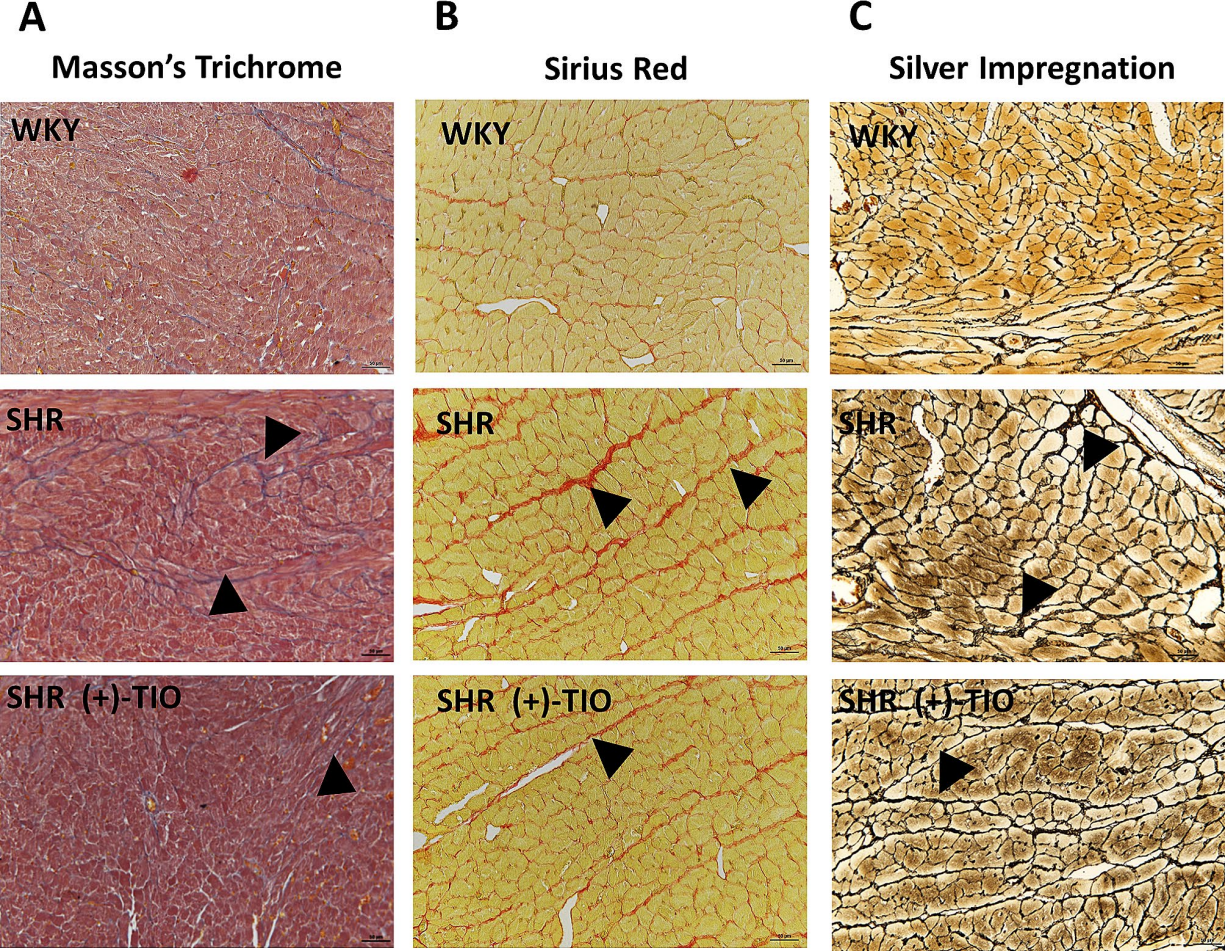
Fibrosis in the heart parenchyma. Cardiac sub-endocardium parenchyma in heart tissue of normotensive Wistar Kyoto rats (WKY), spontaneously hypertensive rats (SHR), and SHR treated with (+)-thioctic acid lysine salt [SHR(+)-TIO] were staining with Masson’s Trichrome technique for connective tissue, with the Sirius red technique to reveal the collagen fibers and with silver impregnation histochemistry to highlighted reticulin fibers. The accumulation of connective tissue fibers was indicated with the black arrowheads. Magnification 20×. Scale bar: 50 μm

Analyses of the myocardium focused at the subendocardial level showed a clear connective tissue accumulation between the cardiomyocytes in SHR rats (Fig. 3A-C), particularly of the reticulin fibers highlighted by the silver impregnation staining techniques (Fig. 3C). Moreover, an increase in cardiomyocytes area was reported in SHR rats compared to WKY rats (Supplementary Fig. 2). These phenomena were reduced by treatment of (+)-TIO, which significantly decreased the cardiomyocytes area (Supplementary Fig. 2) and the left ventricular fibrosis (Fig. 3A-C).

**Fig. 4:**
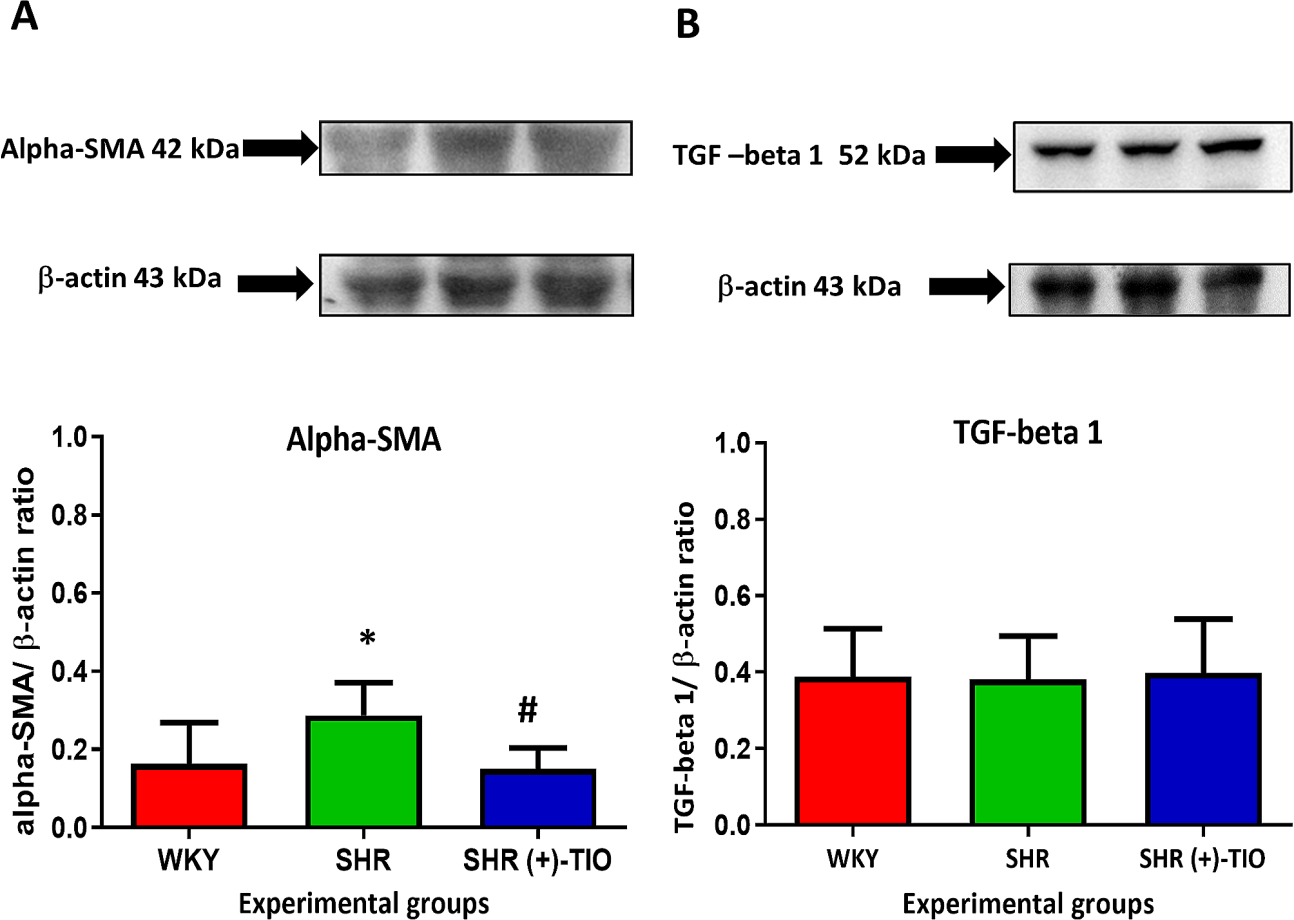
Fibrosis was related to the increase of the alpha-smooth muscle actin (alpha-SMA). Lysates of the heart from normotensive Wistar Kyoto rats (WKY), spontaneously hypertensive rats (SHR), and SHR treated with (+)-thioctic acid lysine salt [SHR(+)-TIO] were immunoblotted using specific antibodies against alpha-SMA (**A**) and transforming growth factor-beta 1 (TGF-beta 1) (**B**). Values indicate the ratio of densitometric analysis of bands and β-actin levels used as the reference loading control. Data are mean ± S.D. *= *p* < 0.05 vs. WKY; #=*p* < 0.05 vs. SHR. Blots are representative of each experimental group

The ventricular fibrosis was related to an increase of the alpha-smooth muscle actin (alpha-SMA) expression (Fig. 4A) in the heart of SHR compared to age-matched WKY, but it was not associated with the modulation of latent form of the transforming growth factor-beta 1 (TGF-beta 1) (Fig. 4B). (+)-TIO was able to decrease the alpha-SMA expression in the heart of SHR (Fig. 4A).

### Inflammation

**Fig. 5:**
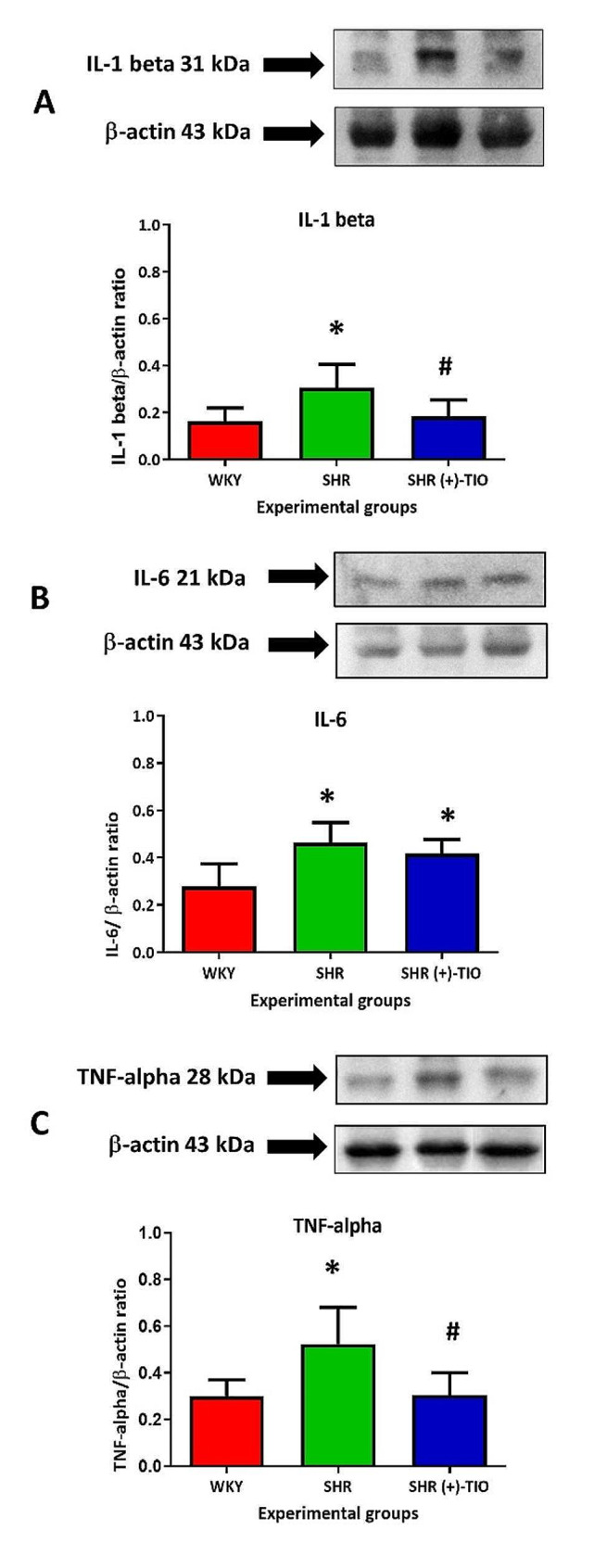
Modulation of inflammatory markers. Lysates of the heart from normotensive Wistar Kyoto rats (WKY), spontaneously hypertensive rats (SHR), and SHR treated with (+)-thioctic acid lysine salt [SHR (+)-TIO] were immunoblotted with specific antibodies against (**A**) interleukin 1 beta (IL-1 beta), (**B**) interleukin-6 (IL-6) and (**C**) tumor necrosis factor-alpha (TNF-alpha). Graphs values indicate the ratio of densitometric analysis of bands to β-actin levels used as the reference loading control. Data are mean ± S.D. *= *p* < 0.05 vs. WKY; #=*p* < 0.05 vs. SHR. Blots are representative of each experimental group

**Fig. 6:**
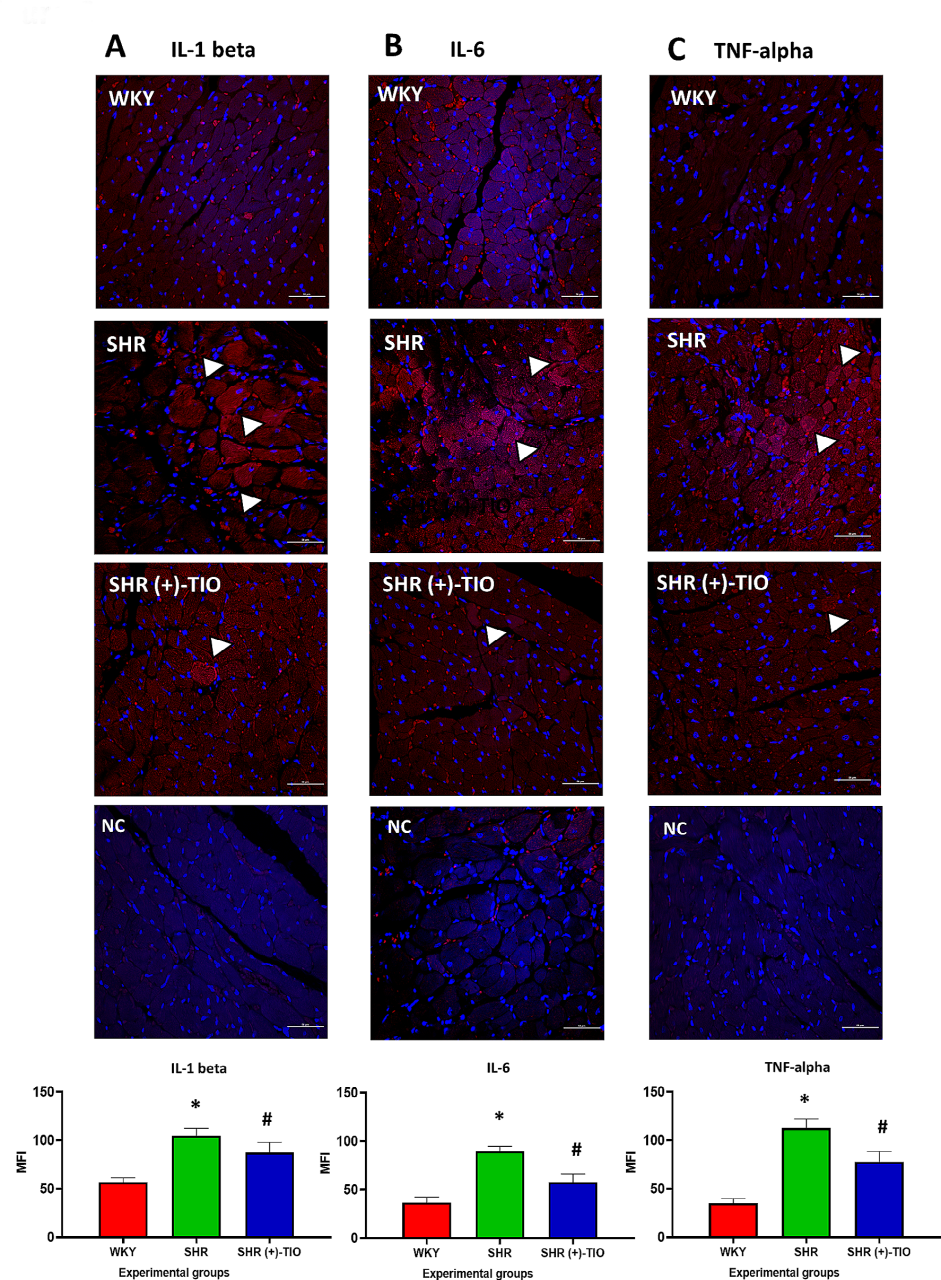
Up-regulation of inflammatory cytokines. Sections of the heart of normotensive Wistar Kyoto rats (WKY), spontaneously hypertensive rats (SHR), and SHR treated with thioctic acid lysine salt [SHR (+)-TIO] processed for the confocal immunofluorescence of (**A**) interleukin-1 beta (IL-1 beta), (**B**) interleukin-6 (IL-6) and (**C**) tumor necrosis factor-alpha (TNF-alpha). The graphs showed the values of mean fluorescence intensity (MFI). The immunoreactive cardiomyocytes are indicated with the arrowheads. Data are mean ± S.D. *= *p* < 0.05 vs. WKY; #=*p* < 0.05 vs. SHR. Magnification 40×, zoom 2. Scale bar: 10 μm. NC, negative control

The expression of the cytokine IL-1beta with a band at 31 kDa was increased in the SHR rats in comparison to the age-matched WKY rats as showed by western blot analysis (Fig. 5A). A decreased expression was induced by the treatment with (+)-TIO (Fig. 5A). IL-6 was revealed with a 21 kDa band (Fig. 5B); its expression was higher in SHR compared to the WKY (Fig. 5B) without significant modification in the (+)-TIO treated SHR (Fig. 5B). TNF-alpha, that was revealed with a band at 28 kDa (Fig. 5C) showed a similar trend of the IL-1 beta. The results of western blot analysis were confirmed by the immunohistochemical analysis for IL-1 beta, IL-6, and TNF-alpha that showed an increase in the immunofluorescence intensities of these cytokines in the SHR’s heart. This phenomenon was reverted by (+)-TIO-treatment (Fig. 6A-C).

Immunochemical analysis performed on samples of heart for the evaluation of the expression of intracellular adhesion molecule-1 (ICAM-1), vascular cell adhesion molecule-1 (VCAM-1) and platelet endothelial cell adhesion molecule-1 (PECAM-1) revealed a band approximately 90 kDa for ICAM-1, 95 kDa for VCAM-1 and 130 kDa for PECAM-1 approximately (Supplementary Fig. 3). The expression of adhesion molecules was significantly increased in heart of SHR (Supplementary Fig. 3). Treatment with (+)-TIO countered VCAM-1 and PECAM-1 but not ICAM-1 expression (Supplementary Fig. 3). Moreover, western blot analysis showed an increase in the expression of endothelial markers E-selectin, with a band of approximately 90 kDa, in the heart of the hypertensive rats compared to the normotensive one and (+)-TIO decreases the expression of this endothelial marker (Supplementary Fig. 3).

**Fig. 7:**
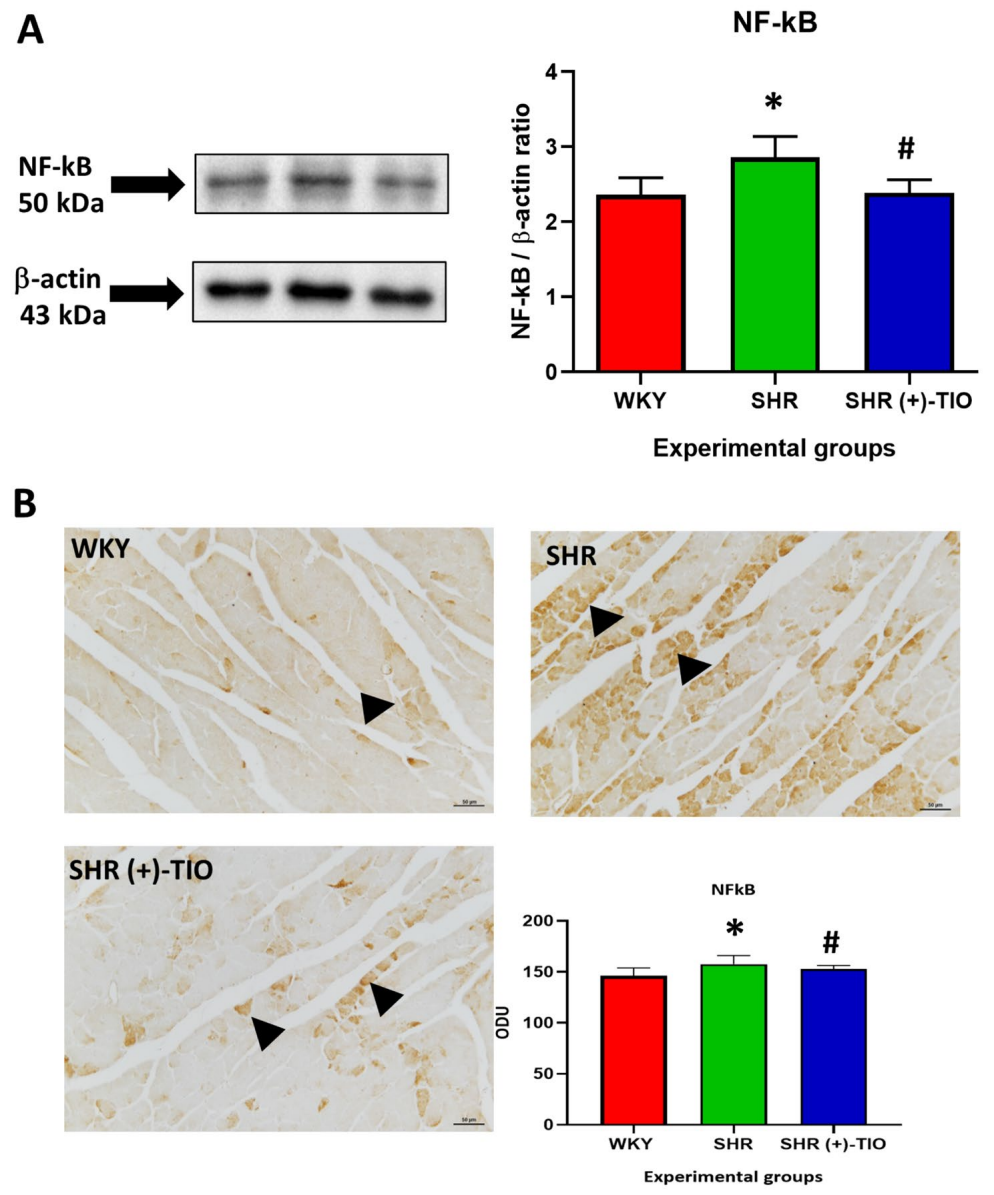
Nuclear factor kappa-light-chain-enhancer of activated B cells (NF-kB) p50 modulated the inflammatory process. Lysates of the heart from normotensive Wistar Kyoto rats (WKY), spontaneously hypertensive rats (SHR), and SHR treated with thioctic acid lysine salt [SHR(+)-TIO] were immunoblotted with specific antibodies (**A**) NF-kB; Values indicate the ratio of densitometric analysis of bands to β actin levels used as the loading control. Data are mean ± S.D. *= *p* < 0.05 vs. WKY; #=*p* < 0.05 vs. SHR. Blots are representative of each experimental group. Sections of the heart (**B**) of WKY, SHR, [SHR (+)-TIO] processed for the immunohistochemistry of NF-kB p50 and quantification in optical density unit (ODU). The immunoreactive cardiomyocytes are indicated with the arrowheads. Magnification 20×. Scale bar: 50 μm

The increased expression of cytokines could be related to the expression of nuclear factor kappa-light-chain-enhancer of activated B cells (NF-kB) p50. As shown in Fig. 7, western blot analysis revealed the elevation of NF-kB levels in the heart of SHR compared to the WKY, modulated by the (+)-TIO treatment (Fig. 7A). This evidence was confirmed by the immunohistochemical analysis (Fig. 7B). The elevated NF-kB immunoreactivity in cardiomyocyte of SHR was reduced in (+)-TIO group (Fig. 7B). Collectively, these results indicated as thioctic acid could reduce the inflammatory process due to hypertension at the cardiovascular level.

## Discussion

Hypertension is a global health problem and is considered the most common risk factor for cardiovascular diseases (CVD). The recent studies describe a link between HBP and inflammation and demonstrate the involvement of oxidative stress in endothelial dysfunction: two of the key processes in the development of hypertension [15].

CVD are complex events with heterogenous pathophysiologic mechanisms in which increased oxidative stress has been viewed as one of the potential common causes. A balance between the presence of ROS and antioxidants is essential for the proper normal functioning of the cells. A variety of CVD is associated, at least partially, with increased production of ROS [39]. ROS constitute both oxygen free radicals, hydroxyl radicals, peroxyl radicals, and non-radicals such as hydrogen peroxide and hypochlorous acid. Endothelial dysfunction caused by oxidative stress and inflammation represents the main factor that causes different CVD [40].

Oxidative stress is implicated, as a major contributing factor, to hypertension development [41]. One hallmark of this damage is endothelial dysfunction, an impairment characterized by a shift in the endothelium with increased vasoconstriction, oxidation, inflammation, thrombosis, and proliferation [42]. However, whether hypertension is the cause of endothelial cell damage is still not clear, although many vascular beds show endothelial damage during hypertension [43]. There are several oxidative-stress-mediated mechanisms involved in the pathogenesis of programmed hypertension, including increased ROS producing enzyme expression, decreased antioxidant capabilities, impaired asymmetric dimethylarginine-NO pathway, increased peroxynitrite, and increased oxidative damage [44].

Campos and coworkers [45] assessed an increase of oxidative stress during compensated cardiac hypertrophy in SHR, with an accumulation of lipid peroxides. Interestingly, their findings demonstrated no accumulation of cardiac 4-HNE protein adducts in SHR. This suggested that the increased 4-HNE generation through lipid peroxidation is counteracted by the increased catalytic activity of aldehyde dehydrogenase 2, the key enzyme in charge of removing intracellular 4-HNE [45]. 4-HNE could also promote chronic inflammation by stimulating the expression of TGFβ in macrophages and smooth muscle cells [46], and, in our study, there were no differences in the 4-HNE and TGFβ expressions in hypertensive rats. We proposed that both were not involved in the inflammatory response.

In the present study, we investigated in SHR the effects of treatment for four weeks with (+)-TIO to analyze the possible protective role of cardiac alterations due to hypertension. As explained, the mechanism of (+)-TIO action is mainly based on its ability to “scavenge” oxygen free radicals and stimulate biosynthesis of reduced glutathione (GSH). (+)-TIO improves antioxidant balance and diminishes oxidative/glycative stress, protein nitrosative damage, inflammation, and apoptosis [47].

Starting from 24 weeks of age, in which the systolic blood pressure is higher in SHR compared to the normotensive WKY, after four weeks of treatment with (+)-TIO, the values of blood pressure were significantly decreased in SHR. This is possibly related to the effects at the levels of the endothelial cells in the vessel. In fact, TIO appears to improve endothelial function through increasing the bioavailability of endothelium-derived NO, decreasing oxidative stress and inflammation [48]. Endothelial cells are important constituents of blood vessels that play key roles in cardiovascular homeostasis [49]. Endothelial dysfunction implicated in the pathophysiology of hypertension is characterized by a reduction of vasodilation, a pro-thrombotic setting, and a pro-inflammatory state. Excessive ROS formation by the vascular wall can mediate these events in the vessels [50]. The vasculature is a major source of NADPH-oxidase-derived ROS, which has a prominent role in vascular damage under pathological conditions [50]. Additionally, endothelium-derived vasoconstricting factors, such as endothelin, urotensin II, vasoconstrictor prostaglandins, angiotensin II, and thromboxane A2, can be released by endothelial cells and contribute to the vasoconstrictor effects. Conversely, reduced NO bioavailability, a well-known endothelium-derived, relaxing factor, is considered a hallmark of endothelial dysfunction [51]. In contrast with our previous results [30, 31], data of the present study, showed that (+)-TIO treatment can reduce systolic blood pressure. This effect could be due to the lower basal blood pressure of the SHR strain used in this study. This evidence may represent an important indication of how a therapy with antioxidants can show antihypertensive properties in the early stages of disease development as suggested by Kizhakekuttu et al. [52]. In an animal model of obesity associated with other diseases, including hypertension, TIO decreased the blood pressure at the standard levels [53]. As was previously reported, the antihypertensive role of TIO depends on initial hypertension, and in the case of early hypertension or non-dramatic hypertension, it is effective as a hypotensive drug [53]. Furthermore, as previously demonstrated, dietary TIO acid supplementation in SHRs lowered the systolic blood pressure, cytosolic [Ca^2+^], blood glucose and insulin levels, tissue aldehyde conjugates, and attenuated adverse vascular changes [54]. TIO has also effects on vascular relaxation in SHR at the level of the aortic smooth muscle cells [55].

The results of this study, supported the previous evidence of a cardiac damage in SHR highlithing the inflammatory process [56–58]. This process, besides mechanical and oxidative stress, related to hypertension leads to end-organ damage, principally due to fibrosis [59]. Concerning this, blood vessel remodeling, excessive matrix deposition, and cardiac hypertrophy become maladaptive responses to abnormal blood flow related to hypertension [60–62]. In accordance, we showed in SHR rats left ventricular hypertrophy and increased fibrosis with deposition of collagen and reticulin fibers. Moreover, the inflammatory pathway and oxidative stress can be triggered by hypertension. Previously, it was demonstrated an increase in lipids peroxidation and nucleic acid oxidation in plasma, kidneys, and hearts of SHR rats [30, 31]. Not only the presence of oxidative stress but also elevated endothelial adhesion molecules such ICAM-1, VCAM-1 and PECAM-1 expression were found in the heart endothelium of hypertensive rats [31]. The present results confirm in the heart of SHR an increased expression of endothelial adhesion molecules related to oxidative stress.

Myocardial injury in long-term hypertension was associated with activation of NF-kB, increased inflammatory cell infiltrate, and an increase expression of the mediators such as IL-1 beta, TNF-alpha, monocyte chemoattractant protein-1, vascular cell adhesion molecule 1, and angiotensinogen [63, 64]. Endothelial cells’ response to NF-kB activation and related inflammation is characterized by the production of adhesion molecules that promote leukocyte adherence and transmigration while also boosting their thrombogenic potential [65]. The vascular wall of SHR has shown an increase in the mRNA expressions of IL-6, IL-1 beta and TNF-alpha. Similarly, increased expressions of other markers of inflammation, including ICAM-1, VCAM-1, monocyte chemoattractant protein (MCP-1) and IL-6, have also been reported in hypertensive rat [66, 67]. In agreement, higher mRNA levels of pro-inflammatory cytokines as well as levels of carbonyl protein were reported in different organs, including the heart, of SHR compared to WKY rats [68].

In the myocardium (+)-TIO showed anti-inflammatory properties, with a reduction of the levels of IL-1 beta and TNF-alpha related to a decrease of NF-kB. A previous study demonstrated that TIO improved cardiac and renal functions, and downregulated the expression levels of IL-1 beta, TNF-alpha, and inducible nitric oxide synthase in the myocardium of septic rats [69]. Furthermore, infection and tissue injury release the pro-inflammatory cytokines, including TNF-alpha, IL-1 beta, and IL-6, which contribute to subsection increased systemic inflammatory responses. TNF-alpha induces an ample range of biological effects, including cell differentiation, apoptosis, and multiple pro-inflammatory effects, which trigger the activation of the NF-kB signaling pathway [70]. Like other studies [71], TIO has been shown to suppress NF-kB activation through direct ROS scavenging or even independent of its antioxidant function [72]. Following these studies, the antioxidant capacity of (+)-TIO is correlated to its anti-inflammatory effect in the animal model of hypertension.

These findings highlighted the importance of antioxidants as supplementary molecules to standard anti-hypertensive therapy. Pre-clinical evidence and clinical randomized studies demonstrated the potential anti-hypertensive effect of antioxidant molecules in the diet both in hypertensive and normotensive subjects [52, 53]. Moreover, prenatal use of natural antioxidants may reverse programming progressions and avoid hypertension of developmental origin [73]. Also, similar dietary approaches showed a reduction in cardiovascular morbidity and mortality in hypertensive subjects [73]. Antioxidant molecules used more frequently include flavonoids, vitamins A, C, and E, L-arginine, and mitochondria‐targeted agents such as TIO, Coenzyme Q10, and acetyl‐L‐carnitine [52]. The in vitro and in vivo properties of TIO have been widely revised [25, 29, 74, 75], in particular, its antioxidant potential as a free radical scavenger, its action as metal chelators and its activity on the repair of oxidized injury and regeneration of natural antioxidants defense, such as glutathione, vitamins C and E [24, 25, 76]. Further important benefits of (+)-TIO supplementation include contributions to mitochondrial metabolic pathways, cell signaling that may increase endothelial nitric oxide synthase (eNOS) coupling, and anti-inflammatory effects [77, 78]. For such reasons, this compound has gained great consideration as an antioxidant in the management of diabetic problems like retinopathy, neuropathy, and other vascular diseases [79]. Moreover, the long-term intermittent treatment with (+)-TIO prevented body weight gain and reduced metabolic and cardiac alterations, corroborating its protective properties on the cardiovascular system [80]. Besides, studies in diabetic rats and other different hypertensive animal models revealed the potential for TIO supplementation to reduce blood pressure [51, 80–82].

## Conclusions

(+)-TIO may be considered as one of the antioxidant candidate molecules for slowing down cardiac alterations as-sociated with hypertension, not only for the prevention the fibrosis but also for the reduction of inflammatory processes. The effects observed with the treatment of (+)-TIO could open new perspectives for a possible coadjuvant care in association with antihypertensive treatment to counteract heart injury, which represents a common feature in hypertensive patients.

### Electronic supplementary material

Below is the link to the electronic supplementary material.

Supplementary Material 1

Supplementary Material 2

## Data Availability

The datasets used and/or analysed during the current study are available from the corresponding author on reasonable request.

## Abbreviations

4-HNE: 4-hydroxynonenal
alpha-SMA: Alpha-smooth muscle actin
ARRIVE: Animal Research: Reporting of In Vivo Experiments
CVD: Cardiovascular diseases
DAPI: 4′6-diamidino-2-phenylindole
eNOS: Endothelial nitric oxide synthase
HBP: High blood pressure
HRP: Horseradish peroxidase
ICAM-1: Intracellular adhesion molecule-1
IHC: Immunohistochemistry
IL-1 beta: Interleukin-1 beta
IL-6: Interleukin-6
MCP-1: Monocyte chemoattractant protein-1
MDA: Malondialdehyde
NF-kB: Nuclear factor kappa-light-chain-enhancer of activated B cells
NIS-Elements: Nikon Image analyzer Software
NO: Nitric oxide
PECAM-1: Platelet endothelial cell adhesion molecule-1
ROS: Reactive oxygen species
SDS-PAGE: Sodium Dodecyl Sulphate - Polyacrylamide Gel Electrophoresis
SHR: Spontaneously hypertensive rats
TBARS: Thiobarbituric-reactive substances
TGF-beta 1: Transforming growth factor-beta 1
TIO: Thioctic acid
TNF-alpha: Tumor necrosis factor-alpha
VCAM-1: Vascular cell adhesion molecule-1
WB: Western blot
WKY: Wistar Kyoto rats
