# Supplementary material for: Protective effects of the R-(+)-thioctic acid treatment: possible anti-inflammatory activity on heart of hypertensive rats
Source: BMC Complement Med Ther. 2024 Jul 24;24:281. doi: 10.1186/s12906-024-04547-6 (PMC11267948; doi:10.1186/s12906-024-04547-6)
Supplement: Supplementary file 1 — Supplementary Material 1 [file 12906_2024_4547_MOESM1_ESM.pdf]

Oxy-blot heart Figure 2 Panel A

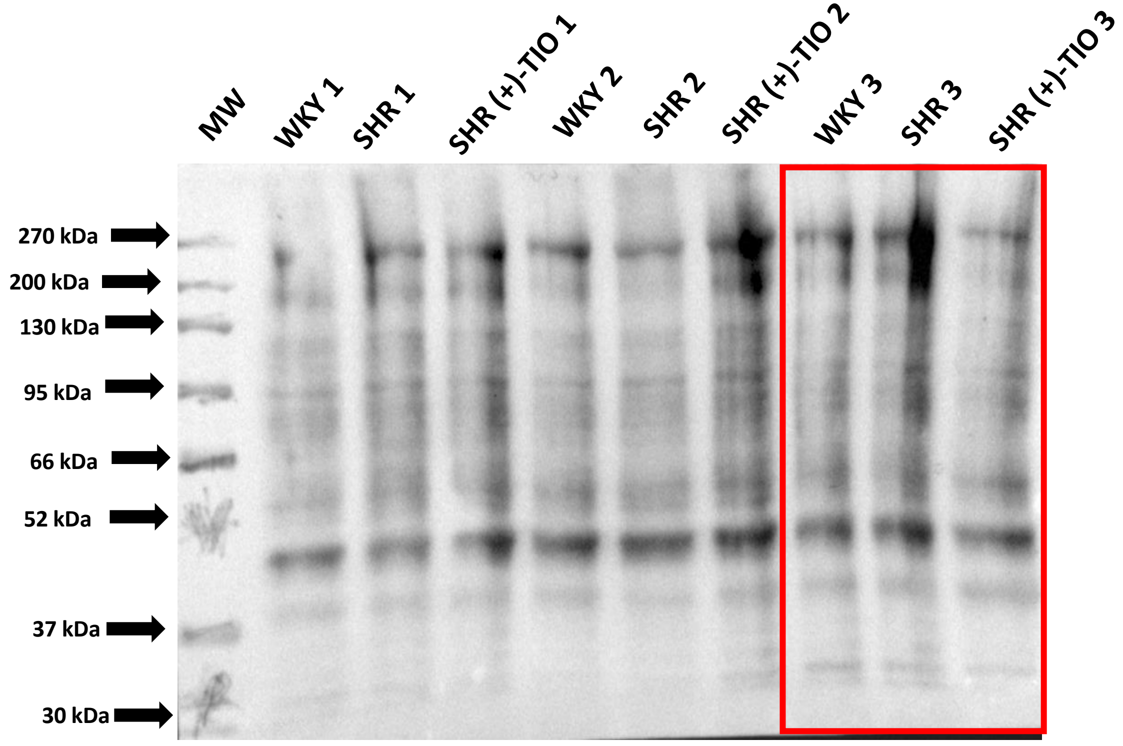

WKY: normotensive Wistar Kyoto rats, SHR: spontaneously hypertensive rats: SHR(+)-TIO: SHR treated with thioctic acid lysine salt; MW: Molecular weight

4-HNE Heart Figure 2 Panel B

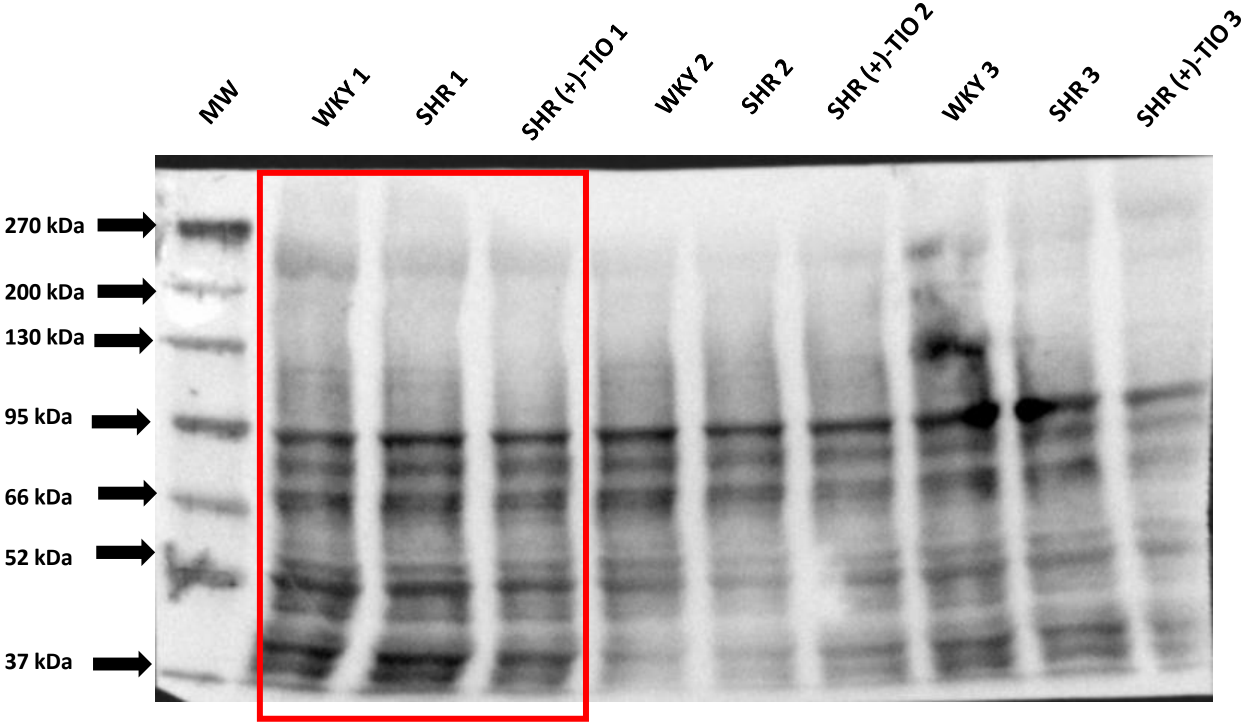

Beta-actin Heart Figure 2 Panel B

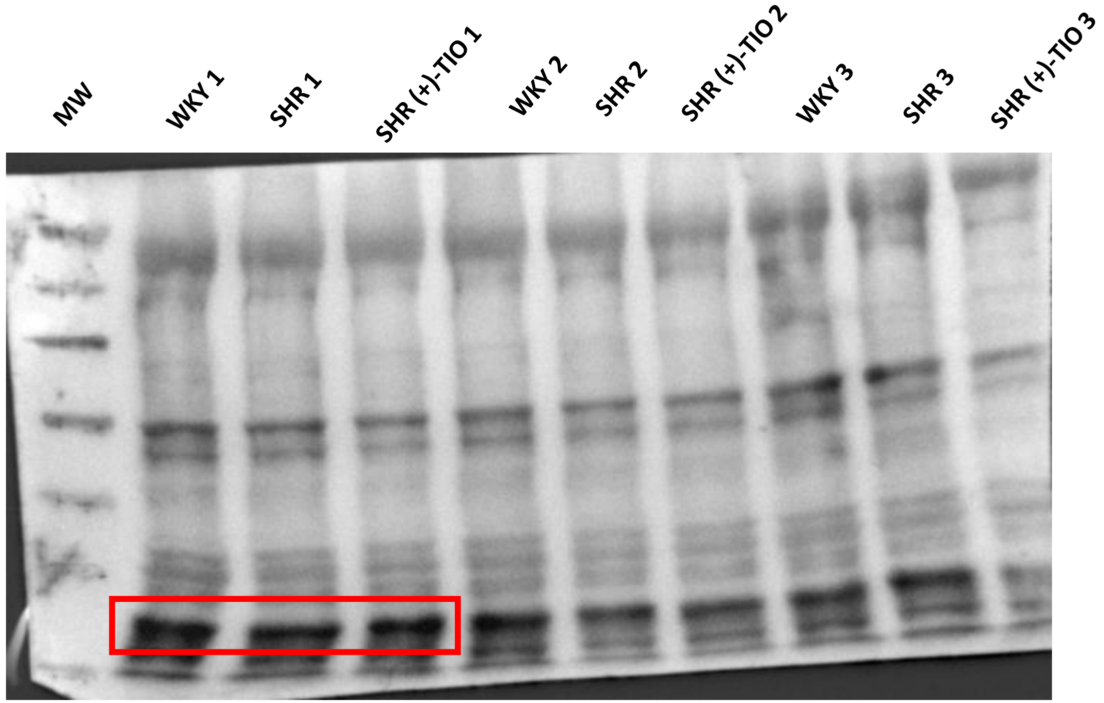

WKY: normotensive Wistar Kyoto rats, SHR: spontaneously hypertensive rats:
SHR(+)-TIO: SHR treated with thioctic acid lysine salt; MW: Molecular weight

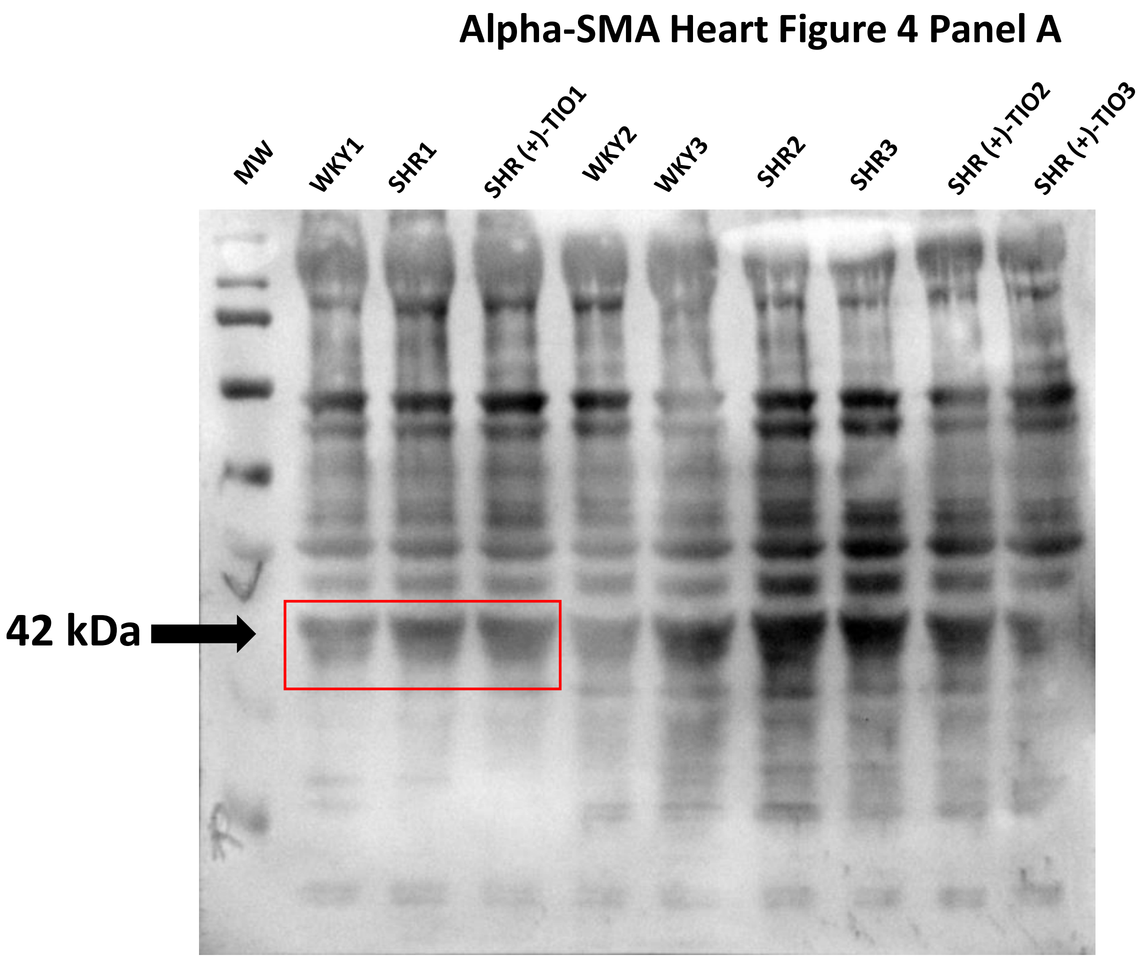

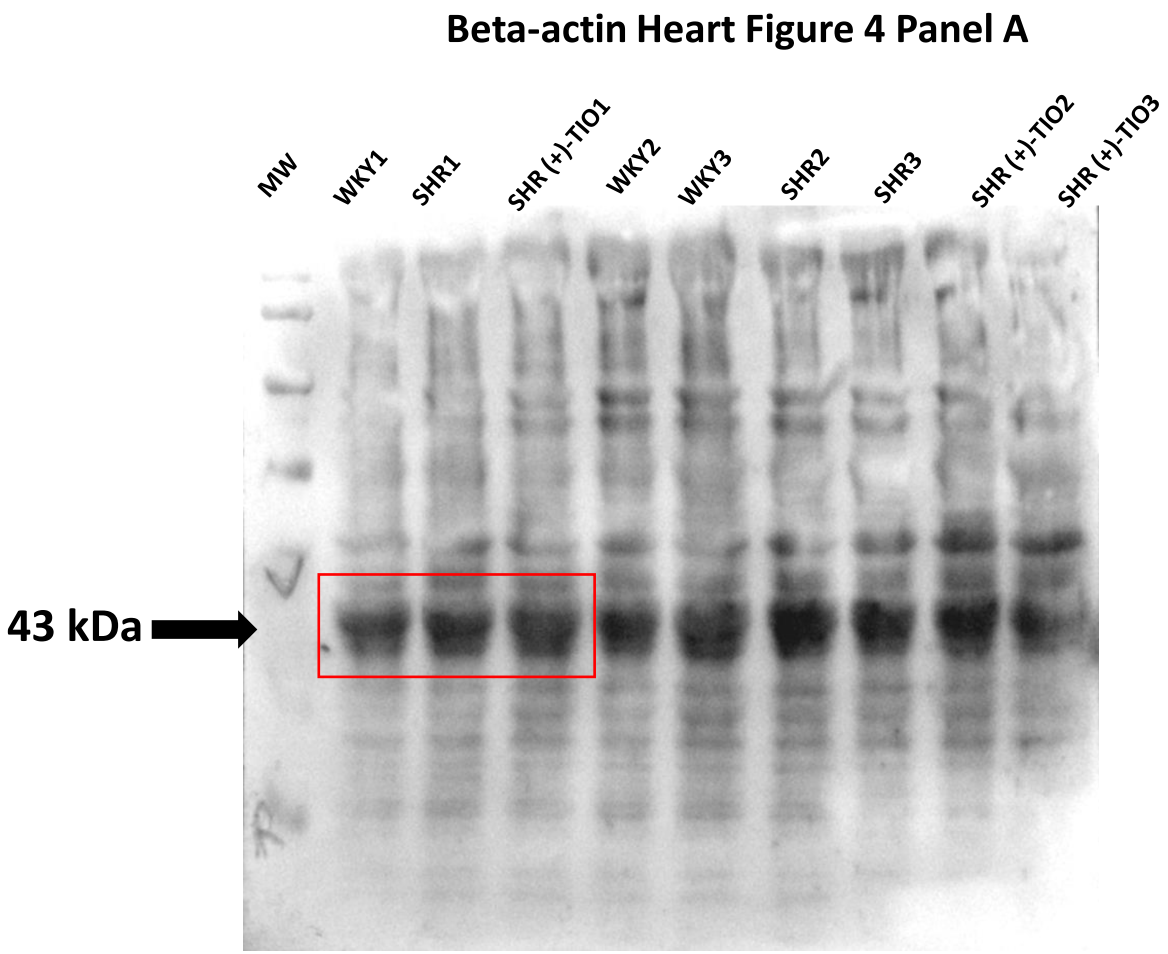

WKY: normotensive Wistar Kyoto rats, SHR: spontaneously hypertensive rats:
SHR(+)-TIO: SHR treated with thioctic acid lysine salt; MW: Molecular weight

TGF-beta 1 Heart Figure 4 Panel B

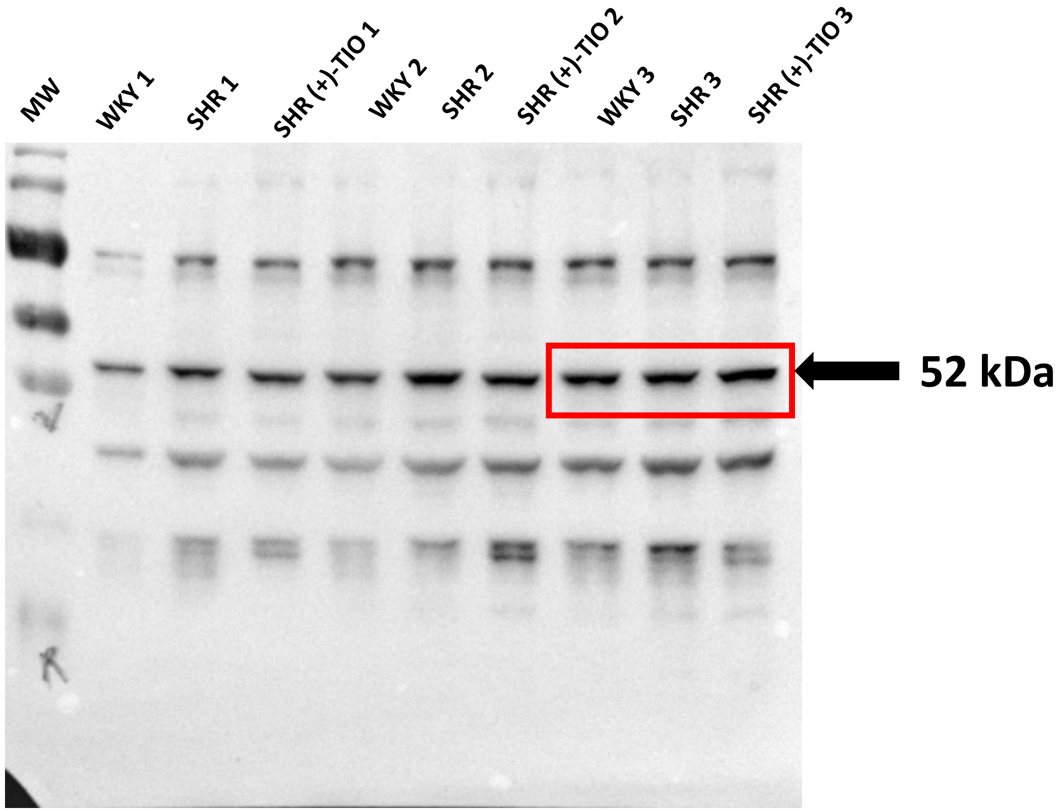

Beta-actin Heart Figure 4 Panel B

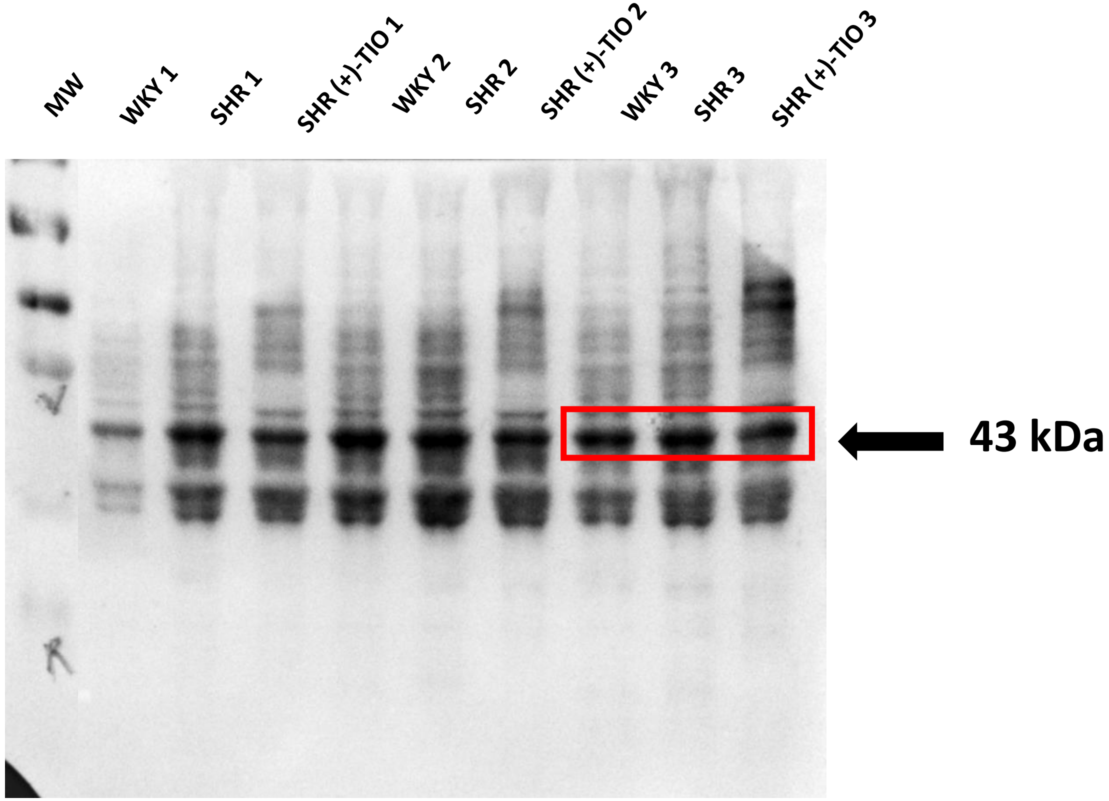

WKY: normotensive Wistar Kyoto rats, SHR: spontaneously hypertensive rats:
SHR(+)-TIO: SHR treated with thioctic acid lysine salt; MW: Molecular weight

IL -1 beta Heart Figure 5 Panel A

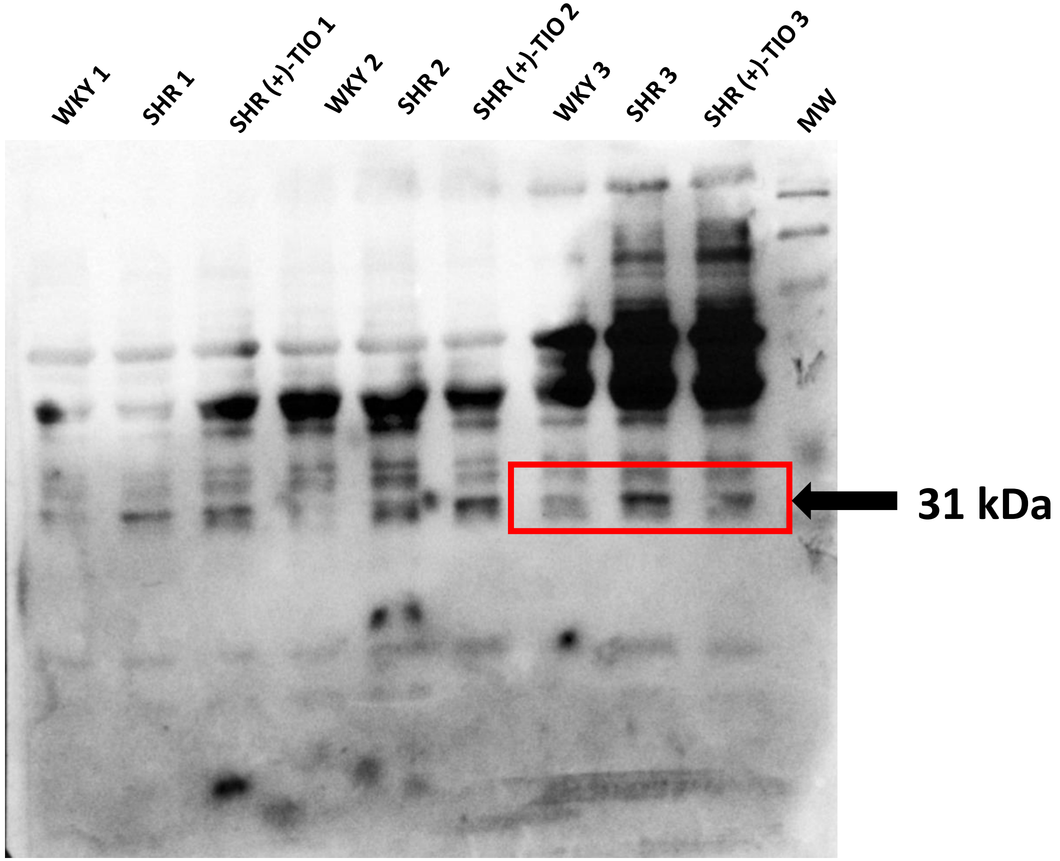

Beta -actin Heart Figure 5 Panel A

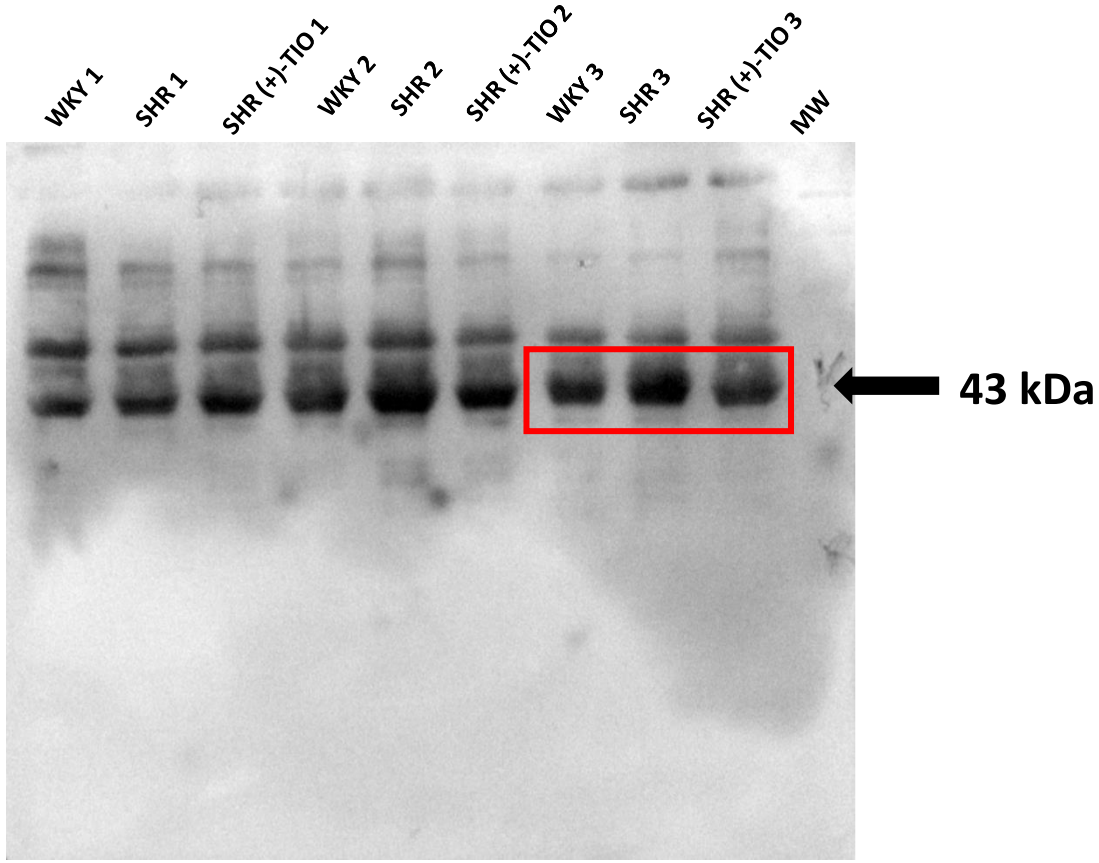

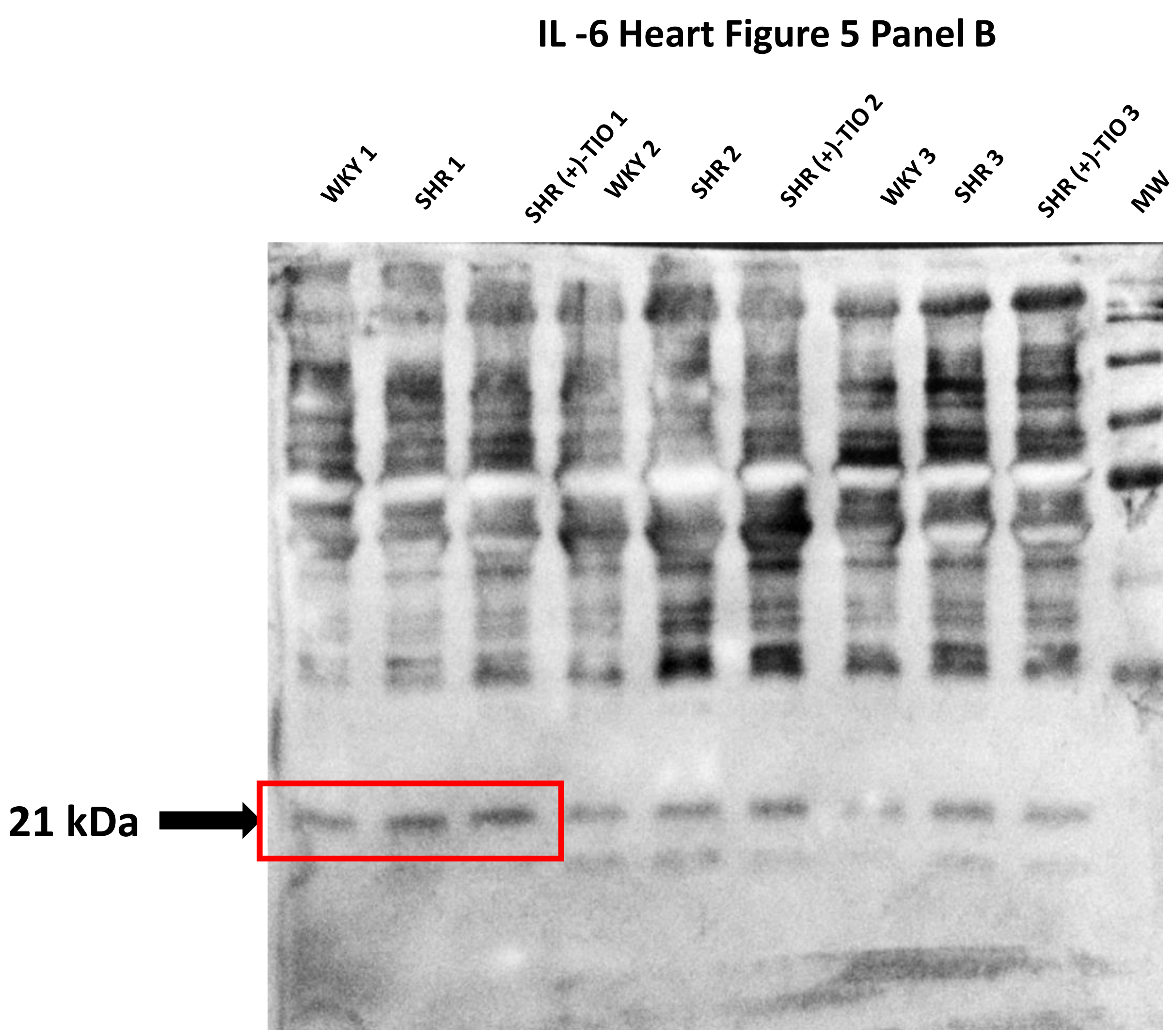

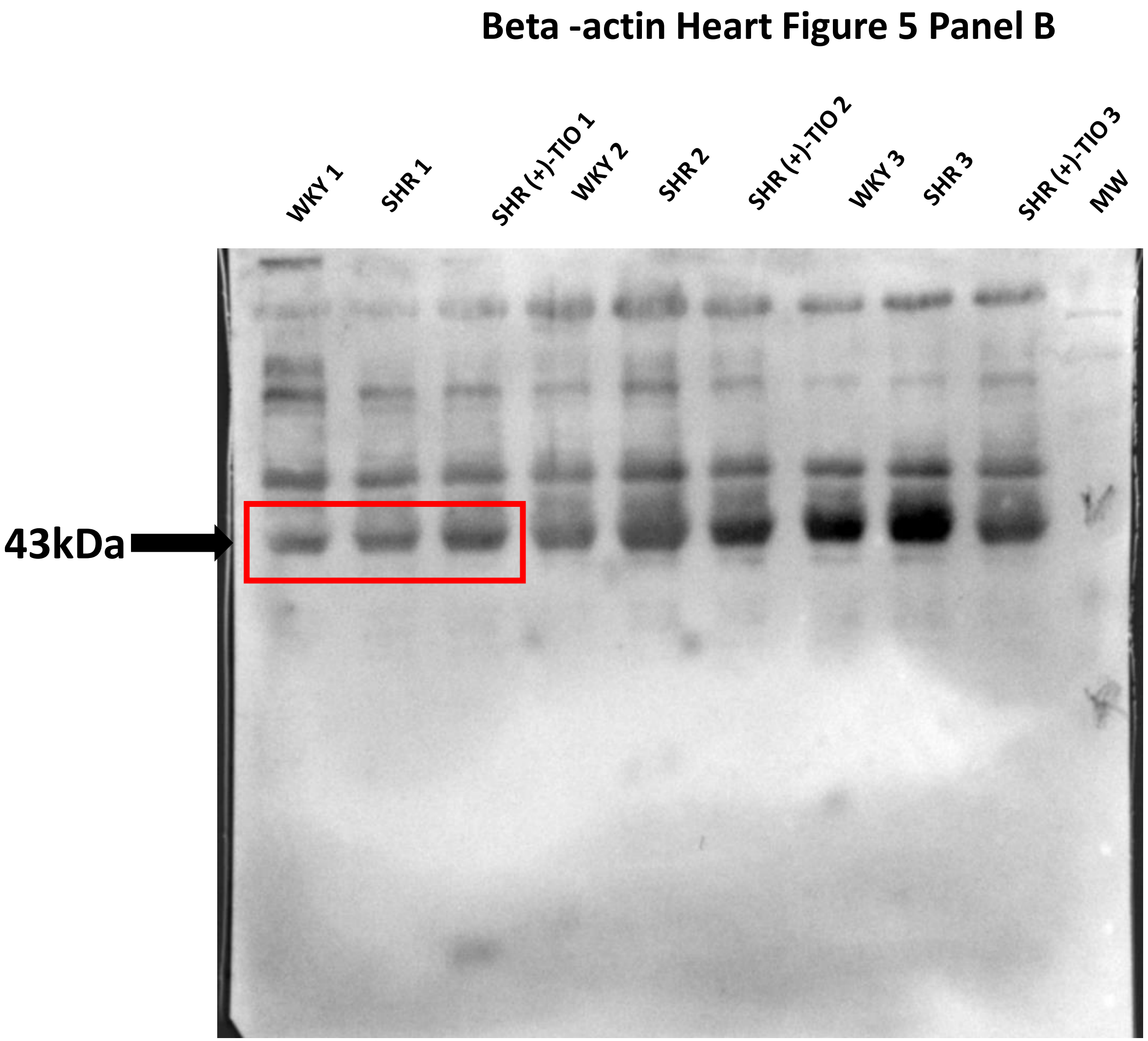

WKY: normotensive Wistar Kyoto rats, SHR: spontaneously hypertensive rats:
SHR(+)-TIO: SHR treated with thioctic acid lysine salt; MW: Molecular weight

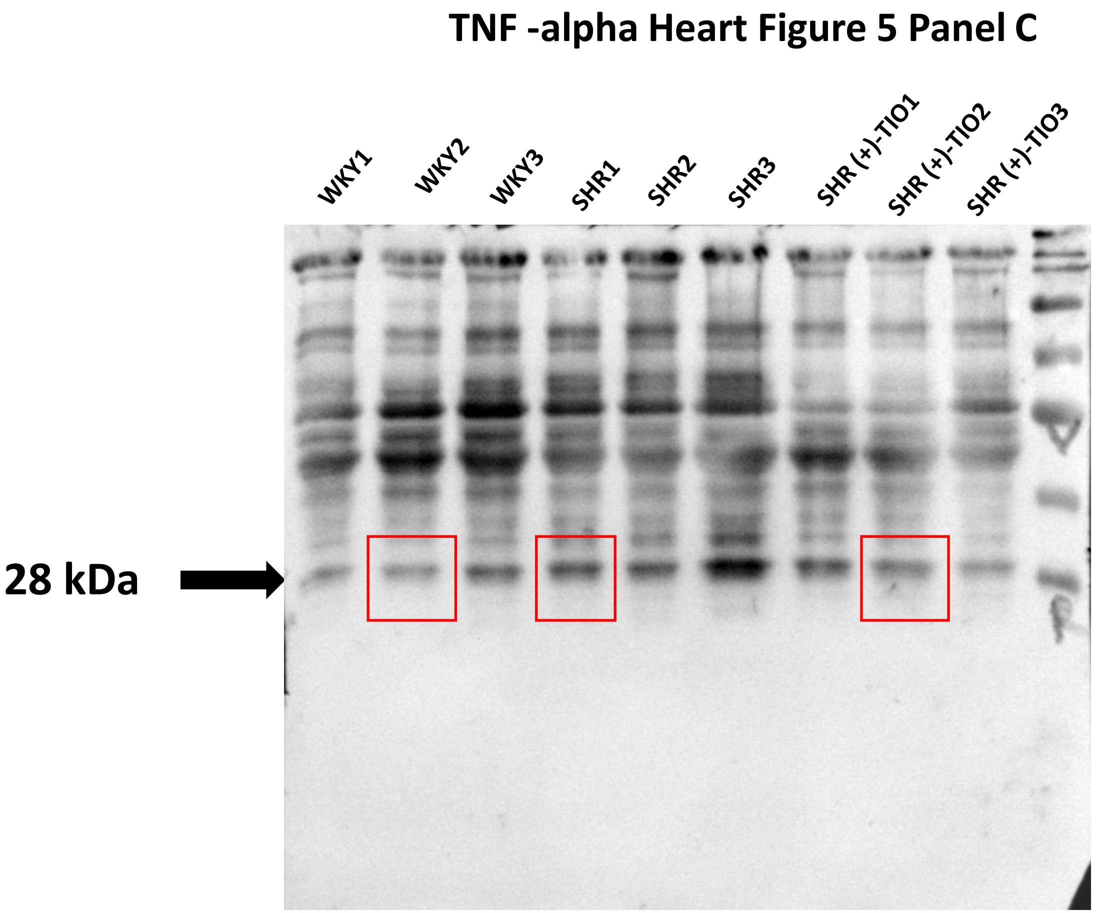

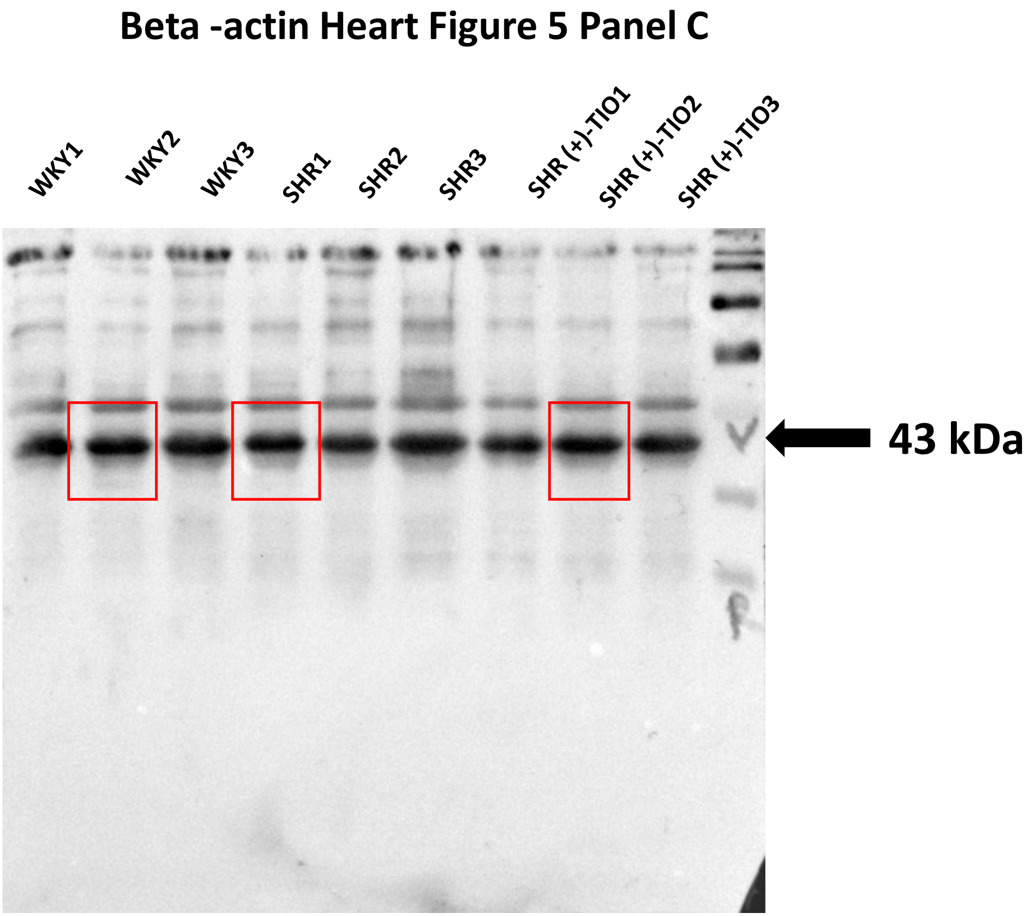

WKY: normotensive Wistar Kyoto rats, SHR: spontaneously hypertensive rats:
SHR(+)-TIO: SHR treated with thioctic acid lysine salt; MW: Molecular weight

NF-kB p50 Heart Figure 7 Panel A

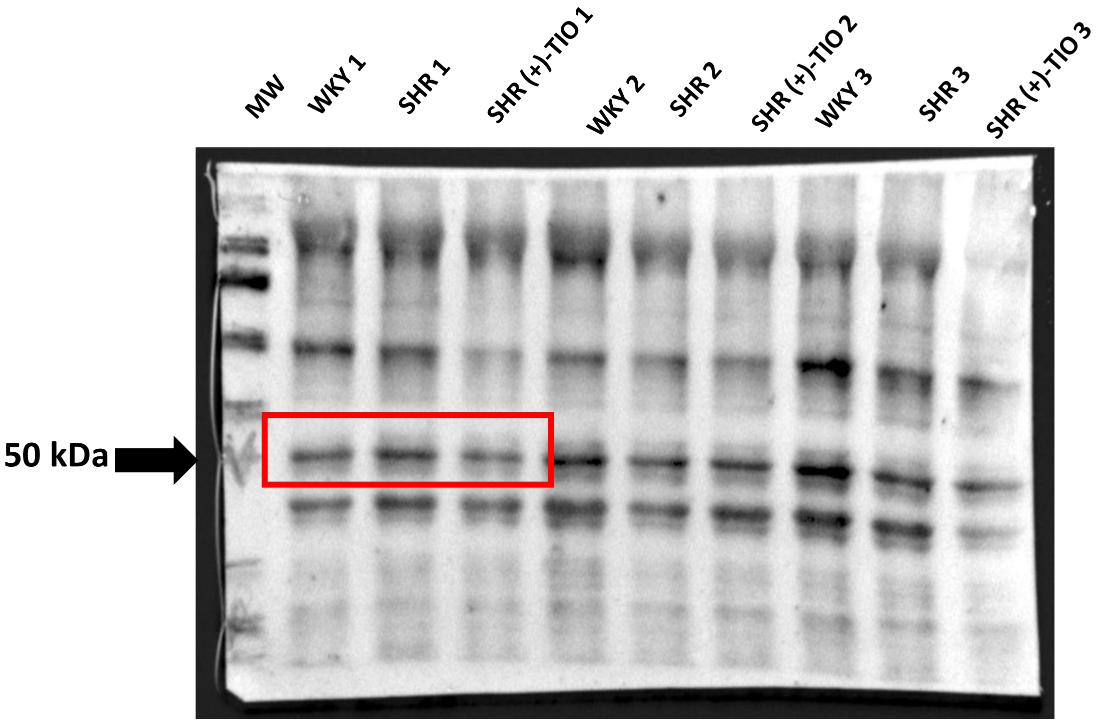

beta-actin NF-kB p50 Heart Figure 7 Panel A

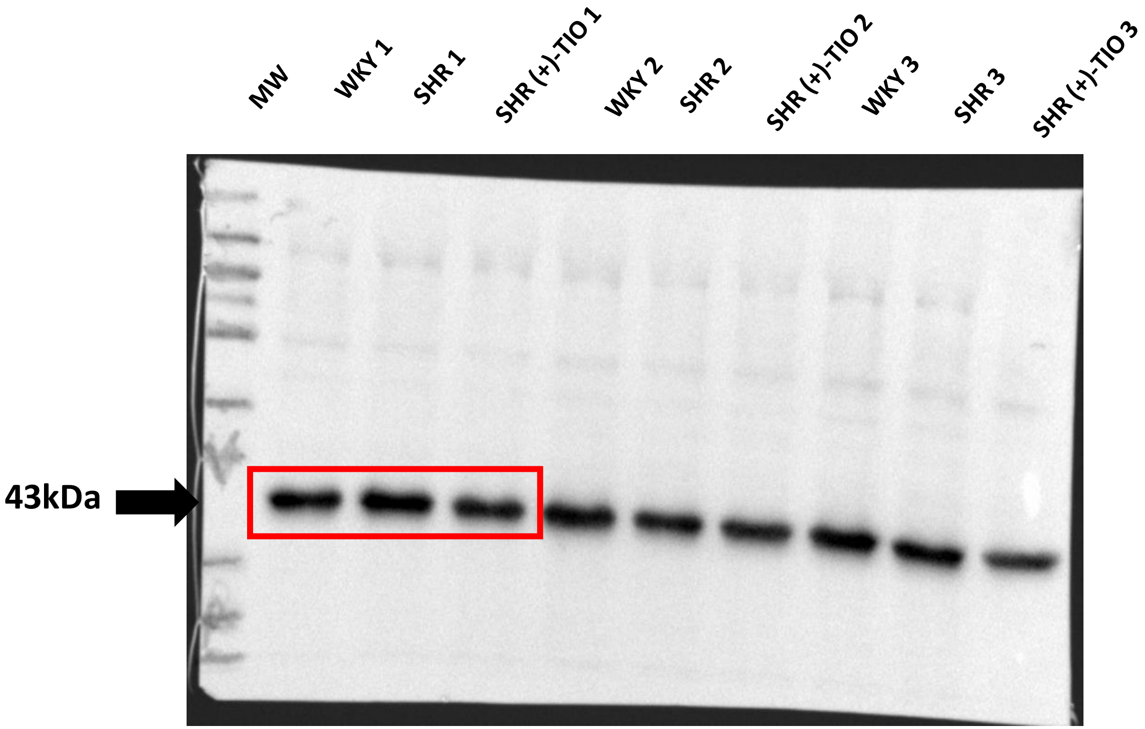

WKY: normotensive Wistar Kyoto rats, SHR: spontaneously hypertensive rats:
SHR(+)-TIO: SHR treated with thioctic acid lysine salt; MW: Molecular weight

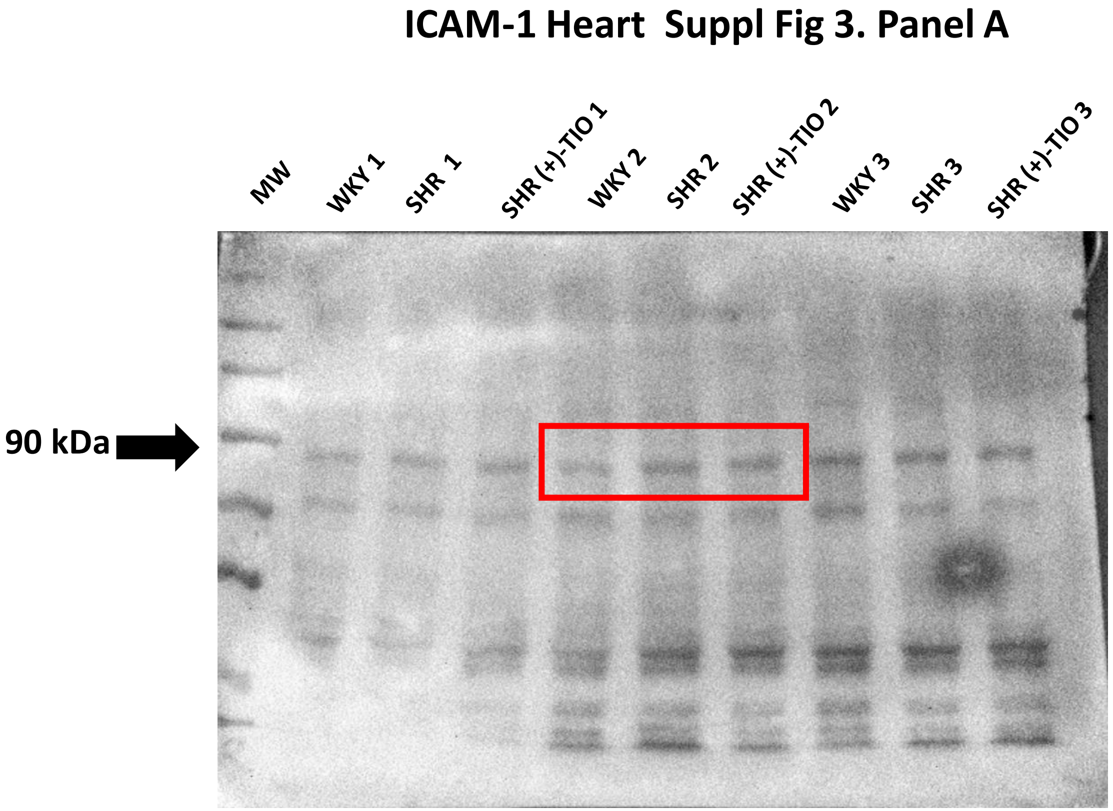

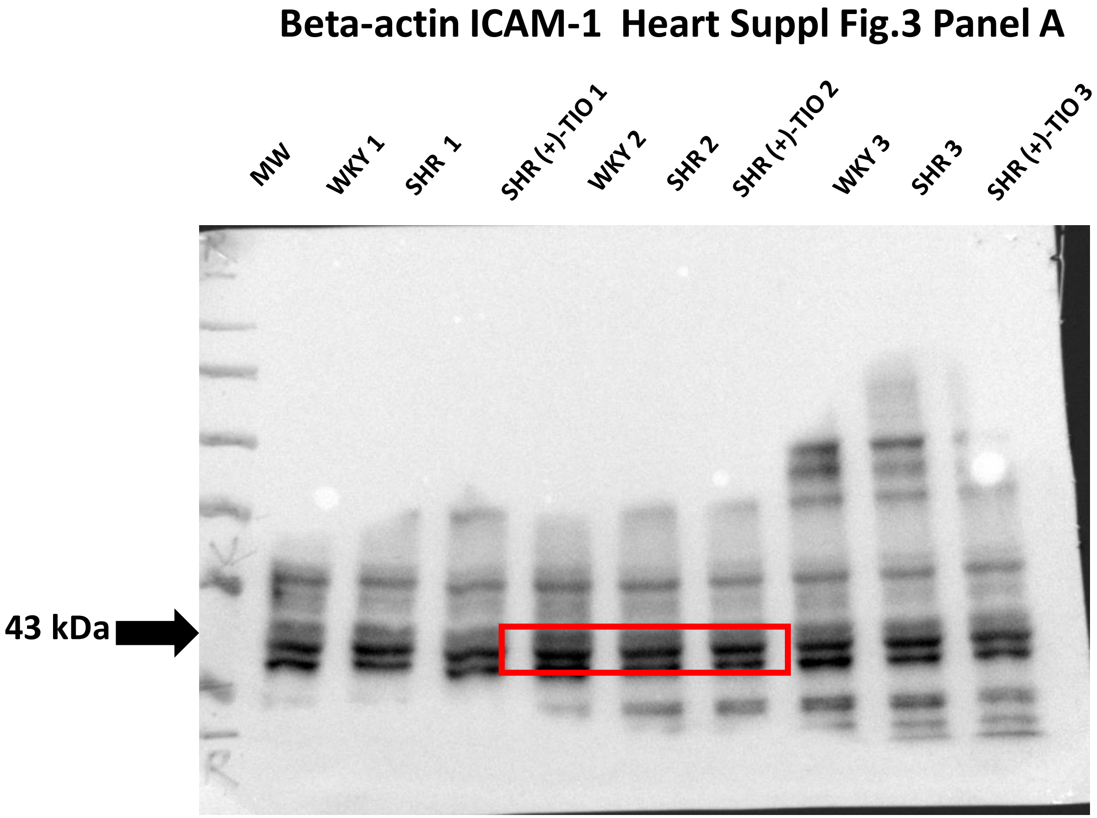

WKY: normotensive Wistar Kyoto rats, SHR: spontaneously hypertensive rats:
SHR(+)-TIO: SHR treated with thioctic acid lysine salt; MW: Molecular weight

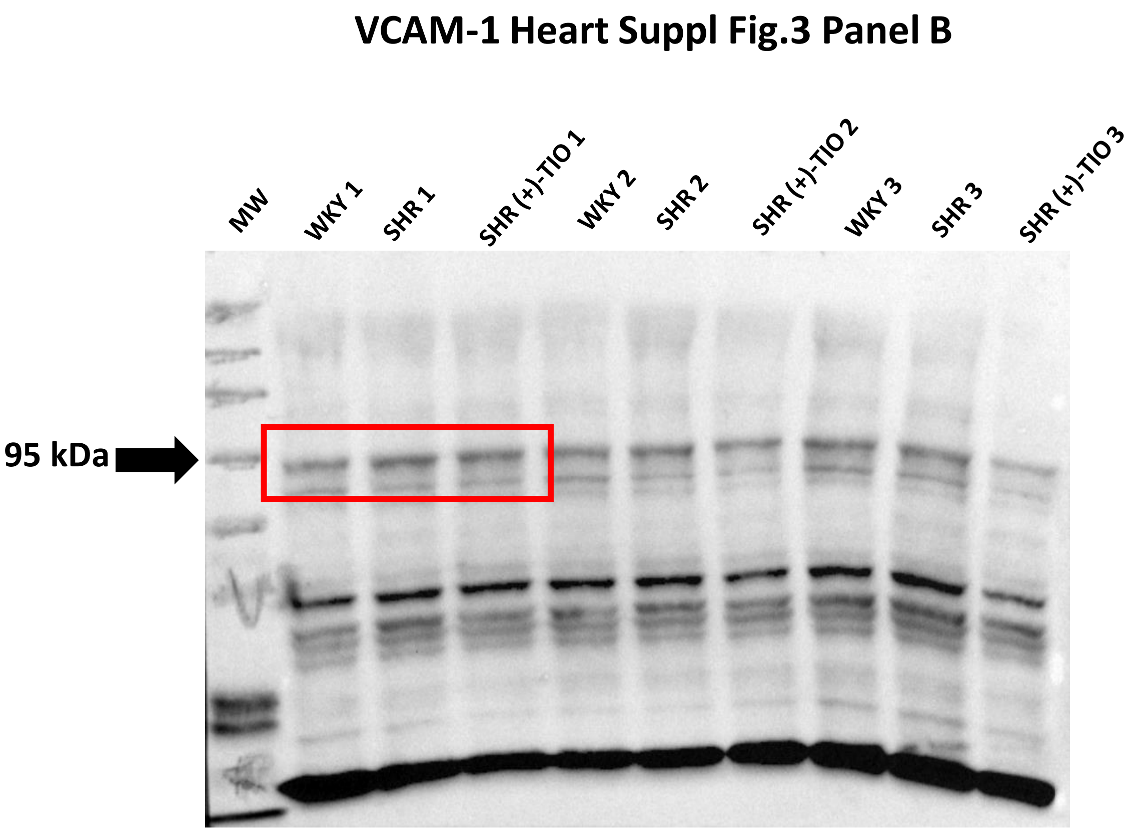

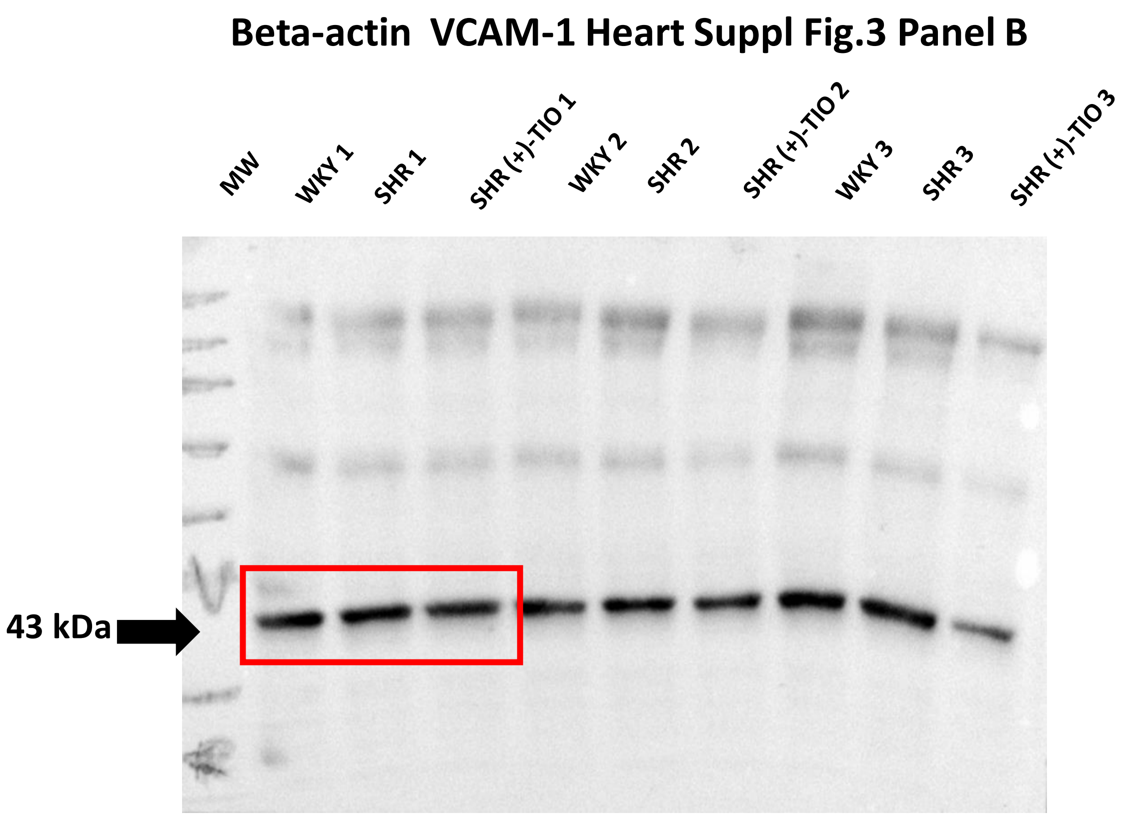

WKY: normotensive Wistar Kyoto rats, SHR: spontaneously hypertensive rats:
SHR(+)-TIO: SHR treated with thioctic acid lysine salt; MW: Molecular weight

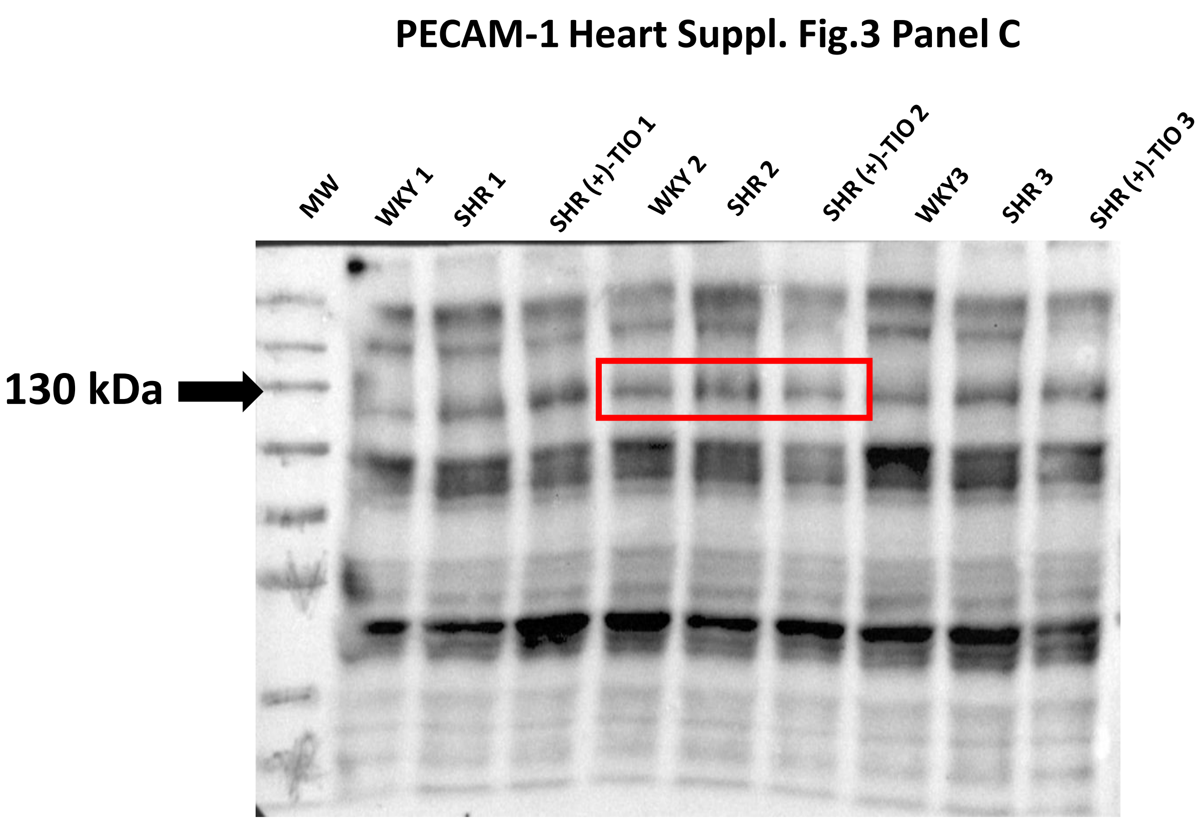

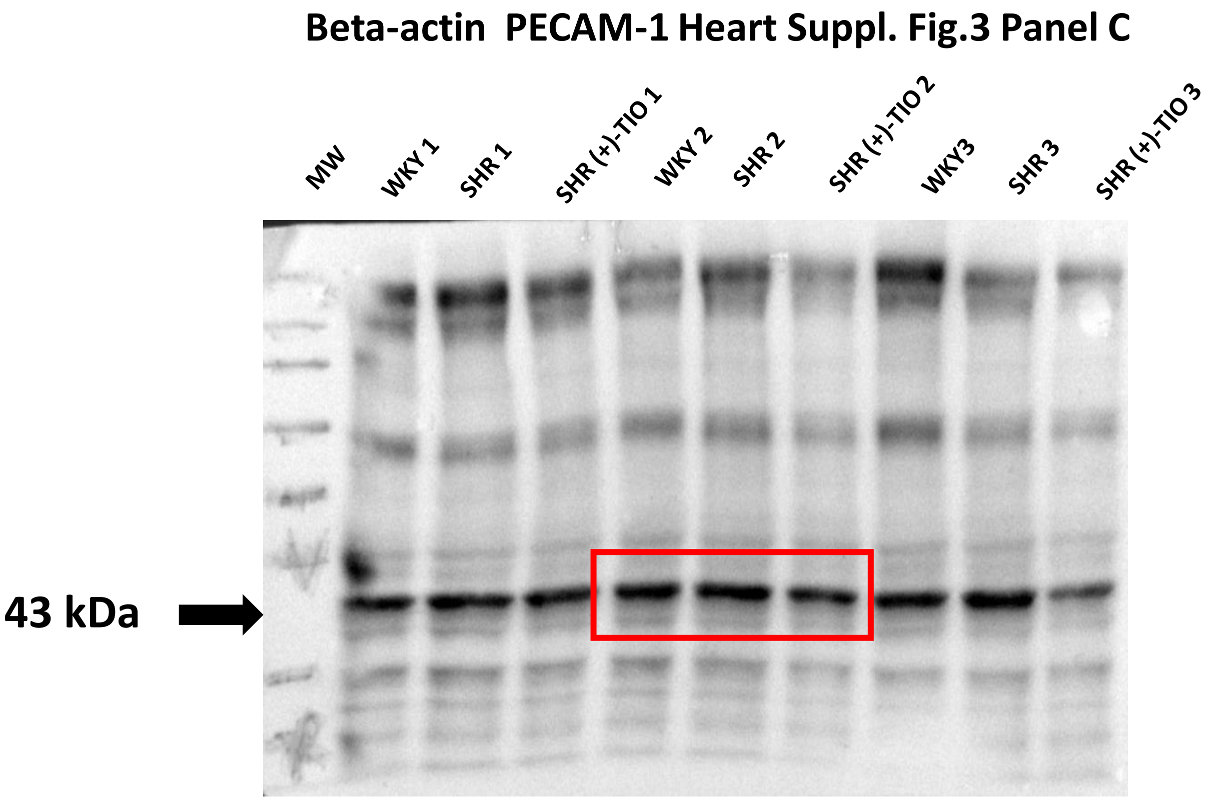

WKY: normotensive Wistar Kyoto rats, SHR: spontaneously hypertensive rats:
SHR(+)-TIO: SHR treated with thioctic acid lysine salt; MW: Molecular weight

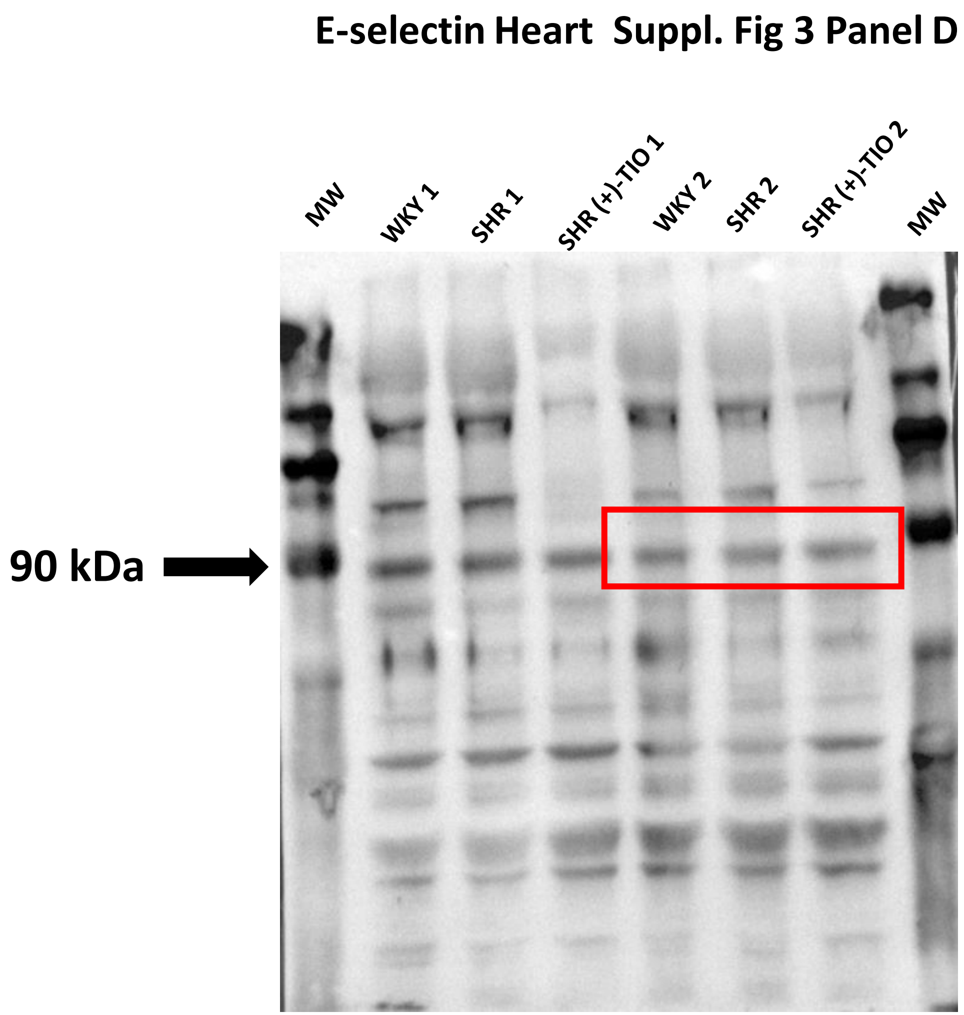

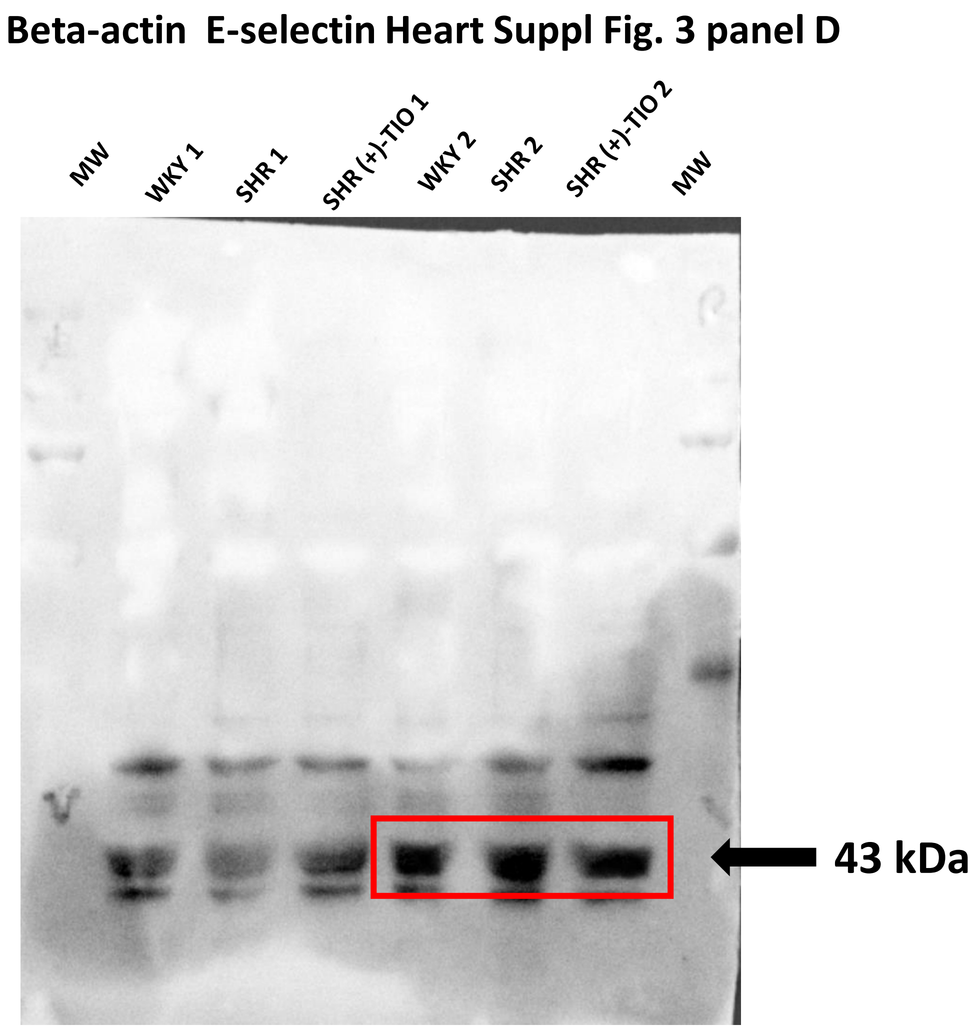

WKY: normotensive Wistar Kyoto rats, SHR: spontaneously hypertensive rats:
SHR(+)-TIO: SHR treated with thioctic acid lysine salt; MW: Molecular weight
