# Supplementary material for: Protective effects of the R-(+)-thioctic acid treatment: possible anti-inflammatory activity on heart of hypertensive rats
Source: BMC Complement Med Ther. 2024 Jul 24;24:281. doi: 10.1186/s12906-024-04547-6 (PMC11267948; doi:10.1186/s12906-024-04547-6)
Supplement: Supplementary file 2 — Supplementary Material 2 [file 12906_2024_4547_MOESM2_ESM.pdf]

Supplementary figure 1

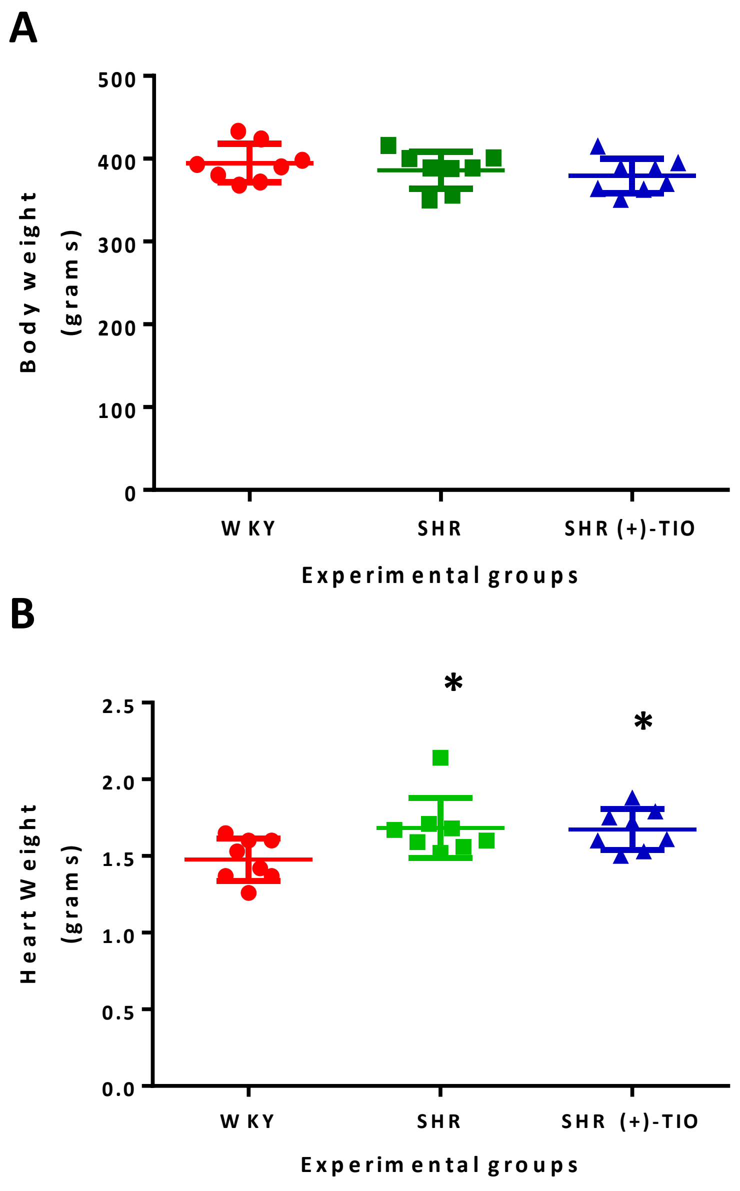

Body weight (A) and heart weight (B) in normotensive Wistar Kyoto rats (WKY), spontaneously hypertensive rats (SHR), and SHR treated with (+)-thioctic acid lysine salt [SHR (+)-TIO]. Data, expressed in grams, are the mean ± S.D. (n=8/group). *= p<0.05 *vs* WKY: #= p<0.05 *vs* SHR.

Supplementary figure 2

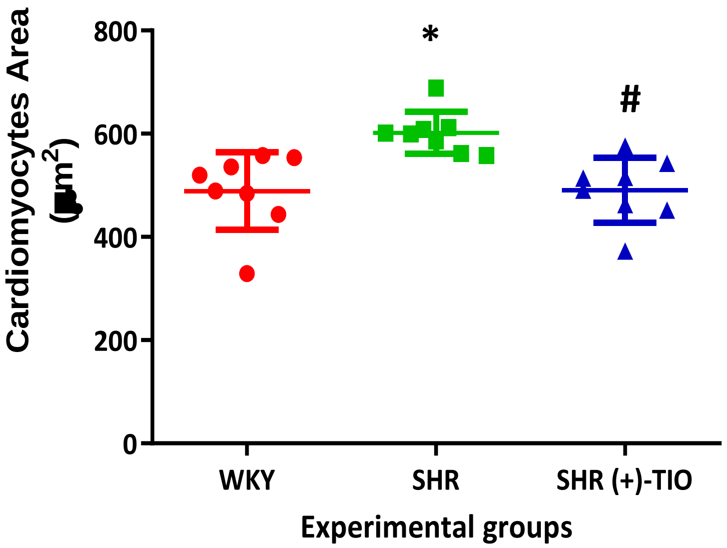

Cardiomyocytes area in normotensive Wistar Kyoto rats (WKY), spontaneously hypertensive rats (SHR), and SHR treated with (+)-thioctic acid lysine salt [SHR (+)-TIO]. Data, expressed in μm^2, are the mean ± S.D (n=8/group). *= $p<0.05$ *vs* WKY: #= $p<0.05$ *vs* SHR.

Supplementary Figure 3

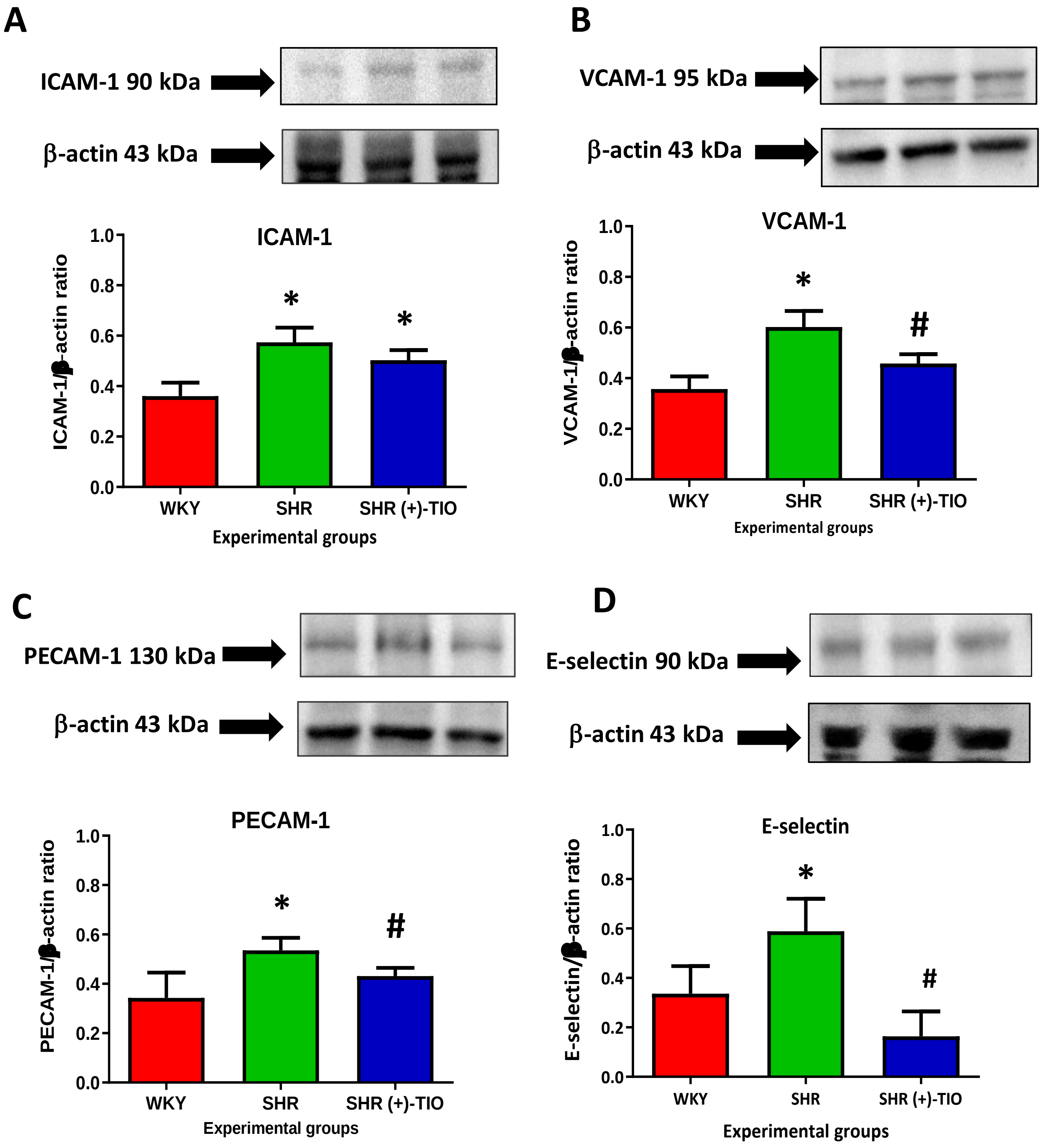

Modulation of vascular adhesion molecules. Lysates of the heart from normotensive Wistar Kyoto rats (WKY), spontaneously hypertensive rats (SHR), and SHR treated with (+)-thioctic acid lysine salt [SHR (+)-TIO] were immunoblotted with specific antibodies against (A) Intercellular Adhesion Molecule-1 (ICAM-1), (B) Vascular Cell Adhesion Molecule-1 (VCAM-1), (C) Platelet endothelial cell adhesion molecule-1 (PECAM-1), (D) E-selctin. Graphs values indicate the ratio of densitometric analysis of bands to β-actin levels used as the reference loading control. Data are mean ± SD *= $p<0.05$ vs WKY; #=$p<0.05$ vs SHR. Blots are representative of each experimental group.
